# B^0^AT2 (SLC6A15) Is Localized to Neurons and Astrocytes, and Is Involved in Mediating the Effect of Leucine in the Brain

**DOI:** 10.1371/journal.pone.0058651

**Published:** 2013-03-07

**Authors:** Maria G. A. Hägglund, Sahar Roshanbin, Erik Löfqvist, Sofie V. Hellsten, Victor C. O. Nilsson, Aniruddha Todkar, Yinan Zhu, Olga Stephansson, Jana Drgonova, George R. Uhl, Helgi B. Schiöth, Robert Fredriksson

**Affiliations:** 1 Department of Neuroscience, Functional Pharmacology, Uppsala University, Uppsala, Sweden; 2 Molecular Neurobiology Branch, National Institute on Drug Abuse, National Institutes of Health, Baltimore, Maryland, United States of America; University of São Paulo, Brazil

## Abstract

The B^0^AT2 protein is a product of the SLC6A15 gene belonging to the SLC6 subfamily and has been shown to be a transporter of essential branched-chain amino acids. We aimed to further characterize the B^0^AT2 transporter in CNS, and to use *Slc6a15* knock out (KO) mice to investigate whether B^0^AT2 is important for mediating the anorexigenic effect of leucine. We used the *Slc6a15* KO mice to investigate the role of B^0^AT2 in brain in response to leucine and in particular the effect on food intake. *Slc6a15* KO mice show lower reduction of food intake as well as lower neuronal activation in the ventromedial hypothalamic nucleus (VMH) in response to leucine injections compared to wild type mice. We also used RT-PCR on rat tissues, *in situ* hybridization and immunohistochemistry on mouse CNS tissues to document in detail the distribution of SLC6A15 on gene and protein levels. We showed that B^0^AT2 immunoreactivity is mainly neuronal, including localization in many GABAergic neurons and spinal cord motor neurons. B^0^AT2 immunoreactivity was also found in astrocytes close to ventricles, and co-localized with cytokeratin and diazepam binding inhibitor (DBI) in epithelial cells of the choroid plexus. The data suggest that B^0^AT2 play a role in leucine homeostasis in the brain.

## Introduction

The essential amino acids leucine and valine cannot be synthesized *de novo* and must therefore be taken up from the diet. Dietary amino acids can enter the bloodstream and thereby reach the brain through mechanisms that include uptake by amino acid transporters [1]–[3], where they are used for energy, in biosyntheses of other molecules and also as direct regulators of brain functions. Dietary leucine has been shown to decrease diet-induced obesity [4] and to activate the mammalian target of rapamycin (mTOR) signalling and to decrease food intake and body weight [5].

The SLC6A15 transporter was identified in 1992 by Uhl *et al*. [6]. This transporter displayed high sequence similarity to neurotransmitter transporters of the solute carrier 6 (SLC6) family [6]–[8]. Commonly used names for the gene product of SLC6A15 include B^0^AT2, SBAT1, NTT73 and V7-3 [9]–[12] and in this paper we will use the notation B^0^AT2. Several reports have shown, using reverse transcription PCR, that SLC6A15 is almost exclusively expressed in the CNS [7]–[9], [12] which is in good agreement with initial *in situ* hybridization data [13]–[15].

The SLC6A15 transporter has been functionally characterized as a Na^+^-coupled amino acid transporter. B^0^AT2 mediates transport of a broad range of amino acids, displaying high-affinity for methionine, proline, and the branched-chain amino acids (BCAAs) valine, leucine and isoleucine [9], [12]. Although B^0^AT2 is fairly well characterized at the biochemical level, the physiological relevance of this transporter is unknown. *Slc6a15* knockout (KO) mice, originally termed v7-3 KO mice [11], are viable, fertile and display behaviours similar to those of wild type (WT) mice. *Slc6a15* KO mice had however a 40% reduction in Na^+^-dependent uptake of leucine, and a 15% reduction in uptake of proline into brain synaptosomes compared to WT mice [11]. Initial work with these KO mice provided support for roles for SLC6A15 in mediating effects of leucine in regulating appetite and food preferences (J. Drgonova, F. Hall, G. Uhl, unpublished observations).

Here we used *Slc6a15* KO mice to investigate the role of B^0^AT2 in brain in response to leucine and in particular the effect on food intake. *Slc6a15* KO mice showed reduced reduction of food intake and lower neuronal activation in the ventromedial hypothalamic nucleus (VMH) in response to leucine injections compared to WT mice. We show that B^0^AT2 mRNA and protein are abundant in neurons and astrocytes in hypothalamus and in other sites of the brain, using immunohistochemistry and *in situ* hybridization. B^0^AT2 immunoreactivity also co-localizes with diazepam binding inhibitor (DBI) and cytokeratin in epithelial cells of the choroid plexus.

## Materials and Methods

### Ethical statement

All animal procedures were approved by the local ethical committee in Uppsala and followed the guidelines of European Communities Council Directive (86/609/EEC).

### Western blot

#### Antibody specificity

We performed a western blot analysis of B^0^AT2 on brain tissue from adult, male C57Bl6/J mice (Taconic M&B, Denmark). The tissue was homogenized in homogenization buffer (50 mM Tris, 150 mM NaCl, 4 mM MgCl, 0.5 mM EDTA, 2% Triton X-100 and 1mM Protease inhibitor PMSF (Sigma-Aldrich, USA) diluted in isopropanol). Protein concentrations were determined by protein assay DC (Bio-Rad, Hercules, USA) according to the manufacturer's instructions. Gel electrophoresis was used to separate the protein lysate (50 µg and 200 µg) together with PageRuler prestained protein ladder (Fermentas, Canada), on a Mini-Protean TGX gel (4–10%, Bio-Rad, Hercules, USA) in running buffer (0.1% SDS, 0.025 M Tris base and 0.192 M glycine). The proteins were transferred to a Immobilon-P polyvinylidene fluoride (PVDF) membrane (Millipore, Billerica, USA) in transfer buffer (0.025 Tris base, 0.192 M glycine and 20% methanol) and pre-blocked for 1 h in blocking buffer (5% non-fat dry milk (Bio-RAD, Hercules, USA) diluted in 0.15 M NaCl, 0.01 M Tris, 0.005% Tween-20, pH 8.0). The membrane was incubated with the custom made polyclonal B^0^AT2 antibody (directed against the peptide sequence: (NH_2_-)DSVEEVSKKSELIVC(-CONH_2_); Table S1) overnight at 4°C. After washes in water, the membrane was incubated for 1 h with horseradish peroxidase conjugated secondary antibody followed by detection with the enhanced chemiluminescent (ECL) method. The membrane was incubated for 3 min in a 1:1 mixture of luminol/enhancer and peroxidase buffer solutions (Immun-Star HRP, Bio-Rad, Hercules, USA) and developed on High performance chemiluminescence film (GE healthcare, Waukesha, USA).

### *In situ* hybridization and immunohistochemistry

#### *In situ* hybridization on free floating sections

Collection and sectioning of mouse brains used for *in situ* hybridization, non-fluorescent and fluorescent immunohistochemistry and *in situ* hybridization were performed as previously described [16]. A probe against mouse *Slc6a15* was synthesized from the mouse *Slc6a15* EST clone ID 6419833 (Invitrogen, USA), See Figure S1 for probe design. A final concentration of 800 ng probe/ml hybridization buffer was used during the method.

#### Non-fluorescent immunohistochemistry on free floating mice sections

Floating sections were processed and either single or double immunohistochemistry was performed. Sections were rinsed in TBS, incubated in 3% H_2_O_2_ and 10% methanol diluted in TBS for 20 min, and again rinsed in TBS. After pre-blocking in 1% blocking reagent (Roche Diagnostics, Switzerland) diluted in TBS, sections were incubated with B^0^AT2 or c-Fos antibodies diluted in supermix buffer (0.25% gelatin and 0.5% Triton X-100 in TBS), and incubated for 24 h (48 h if using c-Fos) at 4°C. For antibody information see Table S1. Subsequently, the sections were rinsed in TBS and incubated for 1 h with biotinylated secondary antibody diluted in supermix and incubated in avidin-biotin complex (1∶800; Vectastain ABC kit, Vector Laboratories, USA). The peroxidase was visualized in black with 0.05% 3, 3′-diaminobenzidine tetrahydrochloride (DAB), 0.35% NiCl and 0.01% H_2_O_2_ after 4–10 minutes incubation. For double immunohistochemistry; after completion of the c-Fos staining, all sections were stained for pS6. The primary pS6 antibody and a biotinylated secondary antibody were used, and 0.35% NiCl was excluded from the DAB solution to obtain brown staining. Sections were mounted on gelatin-coated slides, air-dried over night, dehydrated in ascending concentrations of ethanol, soaked in xylene, and mounted in DPX (Sigma-Aldrich, USA). Images were acquired and analyzed using a Pannoramic midi scanner and the Pannoramic viewer software v.1.14 (3DHistech, Hungary).

#### Fluorescent immunohistochemistry on paraffin sections

Fluorescent immunohistochemistry was performed on paraffin embedded mouse brain sections according to Hägglund *et al.* [16] with following changes. The primary B^0^AT2 antibody was used with antibody markers detecting NeuN, Gad67, pan-cytokeratin, GFAP, synaptophysin and DBI overnight at 4°C. For antibody information see Table S1. Fluorescent staining was analyzed using a fluorescent microscope (Zeiss Axioplan2 imaging).

### RT-PCR on rat tissues

#### Quantitative real-time PCR

The animal handling, tissue collection, cDNA synthesis and the real-time PCR method were performed according to Lagerström *et al.* [17]. The two complete brains were dissected into cross sections. Information about primers used for the housekeeping genes rGapdh, rH3f3B and rRpl19 and the gene of interest *rSlc6a15* is shown in Table S2.

#### Data analysis and relative expression calculations

The data analysis and relative expression calculations were performed according to Lagerström *et al.* [17] with the exception of a different normalization procedure: The gene of interest was normalized to the geometric mean of the expression levels of the rH3f3b housekeeping gene and the normalized quantities were then calculated to the expression relative to maximum (fold decrease) by giving the tissue with the highest expression the value 100% and all other levels were normalized accordingly.

### RT-PCR on leucine supplemented WT mice

#### Animal handling and tissue isolation

Twenty-four adult, male C57BL/6J mice (Taconic M&B, Denmark) were single-housed and kept in type IV standard Macrolon cages under controlled environmental conditions (12 h dark/light cycle, in an air humidity of 55% at 21°C). The animals were randomized into a leucine group and a control group (n = 12/group). Both groups had *ad libitum* access to water and standard chow (Lactamin, Sweden). During 48 hours the water was exchanged for the leucine group, to a drinking solution of 1.5 w/v % leucine. The animals were sacrificed by decapitation 3 h into the light period, and the brain tissues of interest were isolated by dissection. Samples were immersed into RNAlater solution (Ambion, USA) at room temperature for 2 hours, and stored at -20°C until further processed. The RNA preparation and cDNA synthesis were performed as described in ‘*RT-PCR on rat tissues*’.

#### Quantitative real-time PCR on mouse tissue

The RT-PCR was performed as described above with following differences: Primers used for housekeeping genes were *mβ-tubulin*, *mRpl19* and *mActb*, and primers for the genes of interest were *mSlc6a15*, mSlc6a17, *mMtor*, *mRps6*, *mEif4e* and *mDbi*. For primer information see Table S2. The RT-PCR reactions were run in a total volume of 20 µl and contained cDNA synthesized from 25 ng of total RNA. The amplification was performed under following conditions; 50 mM of each primer, 75 mM Tris HCL, 50 mM KCl and 20 mM (NH_4_)_2_SO_4_, 4 mM MgCl_2_, 0.25 mM dNTP, 1∶20 DMSO, 1∶4 SYBR Green (Invitrogen, Sweden) and 20 mU/µl Taq polymerase (Biotools, Spain). The experiments were performed in duplicates, and H_2_O was used as negative control for each primer pair (n = 10–12/group).

#### Data analysis and relative expression calculations

The data analysis and relative expression calculations were performed as described in ‘*RT-PCR on rat tissues*’, with the following differences: The corrected and normalized C_t_-values between leucine-fed mice and control mice were analyzed with Student's t-test, with following significance considerations; *p<0.05, **p<0.01, ***p<0.001.

### Food intake studies on leucine treated Slc6a15 KO and WT mice

#### Animal handling and tissue isolation

In total 120 adult, male WT and *Slc6a15* KO mice on C57BL/6J background [11], were single-housed and kept in type IV standard Macrolon cages under controlled environmental conditions (12 h dark/light cycle with lights on at 07.00, in an air humidity of 55% at 21°C). All animals had *ad libitum* access to water and standard chow (Lactamin, Sweden) unless specified otherwise. The genotype was identified with PCR by using the V73f, J2 and BMP4neo1 primers [11], for primer information see Table S2.

#### Cumulative food intake

Twenty WT mice and twenty *Slc6a15* KO mice were randomly chosen. The mice were single housed for two weeks prior analysis. Animals had an initial BW of 23.49±1.86 g (WT mice) and 23.37±1.43 g (KO mice). Food and animals were weighed daily at 09.00 for one week. The food intake was analyzed by dividing the actual food intake for each animal with the weight of the animal for each day, and the cumulative food intake was then calculated by adding the measured food intake for each day based on 19–20 mice.

#### Food intake after leucine or valine injection

Eighty mice were divided into four groups with twenty mice in each group. The four groups were; WT mice given leucine, *Slc6a15* KO given leucine, WT given valine and *Slc6a15* KO given valine. Mice, 6–7 weeks old, were first accustomed to handling and weighing, once a day for 4 days. On the fourth day the animals were food deprived over night (16 hours), the chow was removed just before the onset of the darkness (17.00). On the fifth day the mice were weighed in the morning (08.30) and intraperitoneally (ip) injected with in total 0.2 ml of either 125 mg/kg leucine (Sigma-Aldrich, USA) or valine (Sigma-Aldrich, USA). Animals had an initial BW (day 4) of 20.09±0.00 g (WT mice) and 20.53±1.37 g (KO mice) before food deprivation, and the body weight after food deprivation (day 5) was 17.87±1.05 g (WT mice) and 17.49±1.26 g (KO mice). Bodyweight and food consumption were measured 2 hours, 4 hours and 24 hours after the injection (Leucine experiment I). After one week the procedure with food deprivation overnight and ip injection with either leucine or valine was repeated as described above, now with six animals in each group. One hour after injection the animals were perfused, as described in ‘*Tissue collection and sectioning*’, and the brains were collected. Free floating tissue sections were made and single (Leucine experiment II) and double (Leucine experiment III) immunohistochemistry was performed on sections from Bregma -0.88 to -1.955. The procedure is described in ‘*Non-fluorescent immunohistochemistry on free floating sections*’.

#### Data and cell count analysis

Leucine experiment I: The weights of the food and the mice were analyzed in time intervals between 0–2, 0–4 and 0–24 hours after the injection and the food intake/body weight ± SD were calculated in Microsoft Excel (Microsoft, USA), based on 20 animals/group. Leucine experiment II and III: The stained sections were scanned and Bregma levels of each section were estimated based on cellular morphology. Brain regions of interest were annotated and c-Fos positive nuclei (leucine experiment II) or co-localization of c-Fos and pS6 positive cells (leucine experiment III) were manually quantified, using the Pannoramic viewer software v.1.14 (3DHistech, Hungary), based on 5–6 mice/group. The averages of positive cells on the left and the right side of the brain (coronal sections) were quantified to analyze the total number of activated neurons (± SEM). Bregma levels and annotated regions correspond to coordinates and annotations both from the brain atlas of Franklin and Paxinos (2007) [18] and from the Allen mouse brain reference atlas [19].

Brain regions evaluated for leucine experiment II were; PVN (Bregma -0.488 to -0.855, average -0.65), VMH (Bregma -1.255 to -1.755, average -1.47), DMH (Bregma -1.455 to -1.755, average -1.59) and Arc (Bregma -1.355 to -1.755, average -1.59). For VMH, 62 sections were used for analysis and in total 4679 cells counted on sections from mice injected with leucine, while 52 sections were analyzed and 2982 cells were counted on sections from mice injected with valine. For DMH 32 sections were analyzed and 8242 cells counted on sections from leucine injected mice, while 7469 cells were counted on 35 different sections from valine injected mice. For Arc, in total 2656 cells were counted on 32 sections from mice injected with leucine, while 26 sections were used for analysis and 1749 cells were counted on sections from mice injected with valine. For PVN, 60 sections were used in the analysis and 6572 cells were counted in mice injected with leucine, while 5968 cells were counted on 61 sections from mice injected with valine. Brain regions evaluated for leucine experiment III were; PVN (Bregma -0.82 to -1.34, average -1.0), VMH (Bregma -1.06 to -1.82, average -1.46), DMH (Bregma -1.34 to -1.82, average -1.65) and Arc (Bregma -1.22 to -2.7, average -1.97). For VMH 643 cells were counted on 18 sections from mice injected with leucine, while in total 913 cells were counted on 19 sections from mice injected with valine. For DMH 12 sections were analyzed and 937 cells counted on sections from mice injected with leucine, while 10 sections were used and 615 cells counted on sections from mice injected with valine. For Arc 878 cells were counted on 28 sections from leucine injected mice, while 530 cells were counted on 22 sections from valine injected mice. For PVN 11 sections were used and 173 cells were counted on the sections from mice given leucine, while 12 sections were used and 189 cells counted on sections from mice given valine.

All statistical calculations were performed using GraphPad Prism v.5 (GraphPad Software, USA). The statistical analysis was done using Student's t-test and values were considered significantly different when *p<0.05, **p<0.01, ***p<0.001.

### Microarray expression analyzes of Slc6a15 KO and WT mice

#### Microarray expression analysis

Seven week old male *Slc6a15* KO mice and WT littermate mice, six mice per group, were used for microarray expression analysis. The mice were sacrificed by cervical dislocation and the brains were isolated by dissection on ice. Mid brain was collected (Bregma level 3.56 to -4.72) and immersed into RNAlater solution (Ambion, USA) for 2 hours at 4°C. Total RNA was extracted using RNeasy midi kit (Qiagen, Netherlands) according to manufacturer's instructions. RNA concentrations were determined using a NanoDrop ND-1000 spectrophotometer (NanoDrop Technologies, USA). The RNA quality was evaluated using the Agilent 2100 Bioanalyzer system (Agilent Technologies, USA) and used for further analysis. In total 250 ng of RNA from each sample was used to generate amplified and biotinylated sense-strand cDNA from the entire expressed genome according to the Ambion WT Expression Kit (P/N 4425209 Rev B 05/2009) and Affymetrix GeneChip® WT Terminal Labelling and Hybridization User Manual (Affymetrix, USA) (P/N 702808 Rev. 1). GeneChip® ST Arrays (GeneChip® Mouse Gene 1.0 ST Array) were hybridized for 16 hours, rotated at 60 rpm, at 45°C. According to the GeneChip® Expression Wash, Stain and Scan Manual (Affymetrix, USA) (PN 702731 Rev 2) the arrays were then washed and stained using the Fluidics Station 450 and finally scanned using the GeneChip® Scanner 3000 7G.

#### Microarray data analysis

The raw data were normalized using the robust multi-array average (RMA) method first suggested by Li and Wong in 2001 [20] utilizing the Affymetrix® Expression Console™ software. Subsequent analysis of the gene expression data was carried out in the freely available statistical computing language R (http://www.r-project.org) using packages available from the Bioconductor project (www.bioconductor.org). In order to search for the differentially expressed genes between the KO and the WT samples, an empirical Bayes moderated t-test was applied [21], using the ‘limma’ package [22]. To address the problem with multiple testing, the p-values were adjusted using the method of Benjamini and Hochberg [23]. A principal component analysis (PCA) was performed in MATLAB (Mathworks, USA) to investigate the correlation between the animals in the two groups, and to study if the mice cluster by genotype. The 400 most differently expressed genes were annotated and biological processes were analyzed using the database for annotation, visualization and integrated discovery (DAVID) (http://david.abcc.ncifcrf.gov) using default settings, with FDR correction for multiple testing [24].

## Results

### Gene expression in Slc6a15 KO mice and WT mice does not cluster by genotype

**Figure 1:**
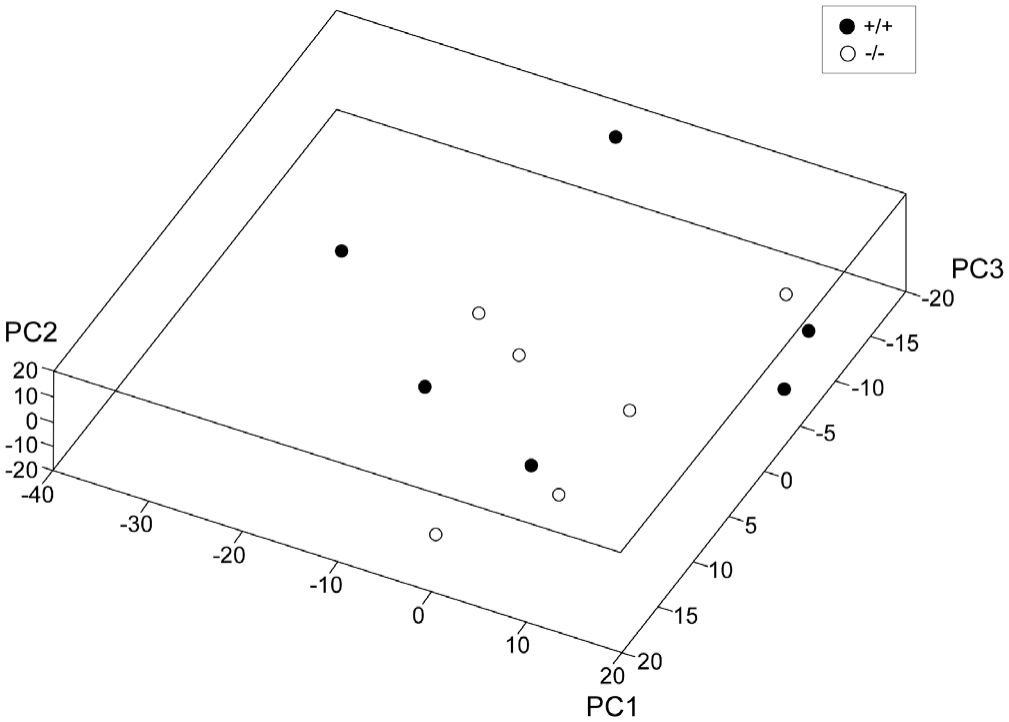
*Slc6a15* KO mice and WT littermates did not cluster by genotype. Gene based principal component analysis (PCA) 3D plot demonstrating microarray gene expression profiling of *Slc6a15* KO mice (−/−) and WT mice (+/+) (n = 6/group) by clustering of the genotypes by 3 principal components (PC1-3 in the graph). Units in graphs are arbitrary units.

Microarray analysis was used to investigate differences in global gene expressions in the brain of *Slc6a15* KO mice compared to WT control mice. The gene-level analysis on a whole-genome scale was quality controlled and normalized prior to cluster comparison and pathway analysis. We carried out PCA analysis to investigate global differences between the genotypes. This showed no clustering of the genotypes by 3 principal components, see Fig. 1. This suggests that the variance among the samples contributed by genotype is relatively minor. Pathway analysis was performed on the 200 most up- and 200 down-regulated differentially expressed genes in *Slc6a15* KOs using DAVID [24]. Genes that were most up-regulated in the first cluster were frequently those involved in protein dimerization activity, DNA binding and transcription regulatory activity. The second cluster showed genes involved in molecular functions, including binding of nucleotides and ribonucleotides, while the third cluster showed that SLC6A15 was involved in binding of different ions, see Table 1. On the other hand, the most down-regulated genes in the fourth cluster were shown to be involved in transcription factor and regulatory activity and DNA binding. The fifth cluster showed peptidase activity, while the sixth cluster showed SLC6A15 to be involved in transmembrane transport and channel activity.

**Table 1:** Pathway analysis of genes up- and down-regulated in *Slc6a15* KO mice.

Cluster (enrichment score)	GO (molecular function) term	FDR	N
200 most up-regulated (Fold change) in the *Slc6a15* KO			
Cluster 1 (0.35)	Protein dimerization activity	8.1E^−1^	4
	DNA binding	1.0E^−2^	7
	Transcription regulator activity	1.0E^−2^	3
Cluster 2 (0.20)	Nucleotide binding	1.0E^−2^	7
	Ribonucleotide binding	1.0E^−2^	3
	Purine ribonucleotide binding	1.0E^−2^	3
	Purine nucleotide binding	1.0E^−2^	3
Cluster 3 (0.00)	Transition metal ion binding	1.0E^−2^	6
	Zinc ion binding	1.0E^−2^	4
	Metal ion binding	1.0E^−2^	7
	Cation binding	1.0E^−2^	7
	Ion binding	1.0E^−2^	7
200 most down-regulated (Fold change) in the *Slc6a15* KO			
Cluster 4 (0.41)	Transcription factor activity	9.7E^−1^	8
	Sequence-specific DNA binding	9.9E^−1^	6
	Transcription regulator activity	1.0E^−2^	9
	DNA binding	1.0E^−2^	12
Cluster 5 (0.34)	Endopeptidase activity	9.9E^−1^	5
	Peptidase activity, acting on L-amino acid peptides	1.0E^−2^	5
	Peptidase activity	1.0E^−2^	5
Cluster 6 (0.19)	Gated channel activity	1.0E^−2^	3
	Ion channel activity	1.0E^−2^	3
	Substrate specific channel activity	1.0E^−2^	3
	Passive transmembrane transporter activity	1.0E^−2^	3
	Channel activity	1.0E^−2^	3

The 400 most differently expressed genes were analyzed using the database for annotation, visualization and integrated discovery (DAVID), with false discovery rate (FDR) correction for multiple testing, showing terms of molecular function according to the gene ontology (GO).

### Leucine injection reduces food intake and activates neurons in VMH in WT mice compared to Slc6a15 KO mice

**Figure 2:**
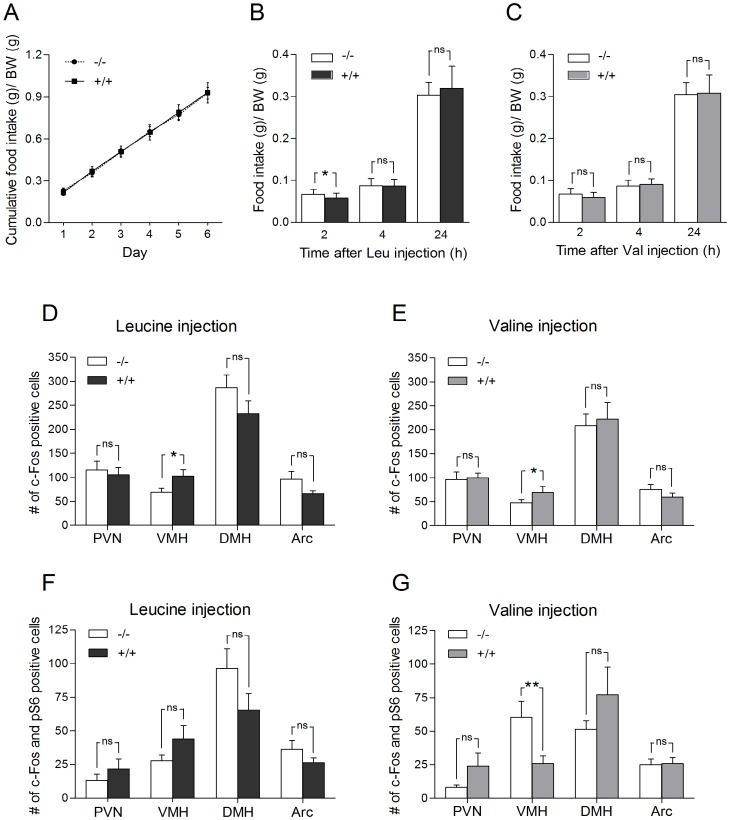
Leucine injections reduces food intake and activates neurons in the ventral medial hypothalamus (VMH) in WT mice compared to *Slc6a15* KO mice. (A) Cumulative food intake of regular chow showed no difference during the 6 days of measurements. Data on the graphs are shown as food intake/body weight (BW) (g) ± SD, for *Slc6a15* KO mice (−/−) and WT mice (+/+) (n = 19–20/group). (B and C) Food intake was analyzed by dividing the actual food intake for each animal with the weight of the animal 0–2, 0–4 and 0–24 hours after leucine (Leu) or valine (Val) injection in *Slc6a15* KO and WT mice (n = 20/group). WT mice given leucine showed a significant (p = 0.0268) reduction of food intake 2 hours after injection. Valine given WT mice showed a decreased but not significant food intake, when measured 2 hours after injection. The food intake after 4 hours or 24 hours was not significantly different between the two groups. (D and E) Number of activated neurons ± SEM (c-Fos positive cells) in food related brain regions after leucine or valine injections in *Slc6a15* KO and WT mice (n = 6/group). WT mice had a significant leucine and valine induced increase (p^Leu^ = 0.0350, p^Val^ = 0.0319) of c-Fos expression in VMH. Both leucine and valine injection gave trends of reduced activation of c-Fos in Arc in WT mice. WT mice had a non-significant reduction of c-Fos expression in dorsal medial hypothalamus (DMH) after leucine injection, a reduction not seen after valine injection. No difference in c-Fos activation was seen in paraventricular nucleus of hypothalamus (PVN) after leucine or valine injection. (F and G) Number of activated pS6 positive neurons ± SEM (c-Fos and pS6 positive cells) in brain regions after leucine or valine injections in *Slc6a15* KO and WT mice (n = 5–6/group). Valine induced significant reduction (p = 0.0096) of activated neurons in the mTOR pathway in VMH in WT mice. Leucine injection non-significantly increased the number of c-Fos and pS6 positive cells in VMH in WT mice, reduced the number of cells co-labelled with c-Fos and pS6 in arcuate nucleus of hypothalamus (Arc) in WT mice, while valine gave no difference between the two groups. Both leucine and valine injection gave trends of increased activation in PVN in WT mice. Leucine injections decreased while valine injections increased the trends of activation of c-Fos and pS6 positive cells in DMH in WT mice. Asterisks denote significance of Student's t-test for difference of means (*p<0.05, **p<0.01, *** p<0.001).

The cumulative food intake of standard chow was measured during one week in both WT and *Slc6a15* KO mice, showing no differences in food intake between the two groups (Fig. 2A). B^0^AT2 functions as an amino acid transporter with high affinity for both leucine and valine [9], [12] and to investigate whether the B^0^AT2 transporter plays a role in mediating a reduction of food intake, we injected leucine or valine intraperitoneally in mice and measured the food intake (leucine experiment I), activation of neurons (leucine experiment II), and activation of neurons in the mTOR pathway (leucine experiment III) in food related brain areas.

Leucine has been shown to reduce food intake in a dose dependent manner with the highest effect 20 min after injection into the mediobasal hypothalamus [25], [26]. In our experiment food intake was analyzed by dividing the actual food intake for each animal with the weight of the animal 0–2, 0–4 and 0–24 hours after injection. A significant reduction in food intake 2 hours after leucine injection (leucine experiment I) was seen in WT mice compared to *Slc6a15* KO mice (p = 0.0268) (Fig. 2B). No differences in food intake were seen after 4 hours (p = 0.8501) or 24 hours (p = 0.2530). Fig. 2C shows that valine caused a non-significant decrease (p = 0.0502) in food intake in the WT mice compared to the *Slc6a15* KO mice, measured 2 hours after injection. Food intake after valine injection did not differ significantly between the two groups after 4 (p = 0.3555) or 24 hours (p = 0.7623). The data are consistent with the idea that the B^0^AT2 transporter plays a role in the uptake of the essential amino acid leucine into the brain and in relaying information of the ventricular leucine concentrations to neurons in the hypothalamus.

To investigate if leucine and valine influence neurons in food related areas, c-Fos immunoreactivity was used as a surrogate marker for neuronal activation. c-Fos expression has been shown to be delayed 30–45 minutes after the stimulus is delivered [27]. To make sure that activation of neurons in the brain could be detected, brains were collected 1 hour following injections. In leucine experiment II, cells were stained with c-Fos antibody to analyze the activation of neurons in hypothalamus after either leucine or valine injection in *Slc6a15* KO and WT mice. Two outliers were found assuming normally-distributed data using Grubbs' test (GraphPad, USA), and excluded from the data set before statistical analysis. The remaining WT mice had a significant leucine-induced increase (p = 0.0350) in c-Fos expression in VMH compared to the *Slc6a15* KO mice (Fig. 2D). Leucine showed weak trends towards reduced c-Fos activation in WT mice in Arc (p = 0.1258) and in DMH (p = 0.1659) nuclei. There was no trend toward activation in PVN (p = 0.6828), compared to *Slc6a15* KO mice. A similar pattern was seen after valine injections. There was a significant valine-induced increase of c-Fos expression in WT in VMH (p = 0.0319) compared to the *Slc6a15* KO mice (Fig. 2E). Valine injections gave a small, non-significant, reduction of activation of c-Fos in Arc (p = 0.2436) in WT compared to *Slc6a15* KO mice, and no difference in c-Fos activation in PVN (p = 0.8428) or DMH (p = 0.7381).

Leucine injections have been shown to induce phosphorylation and activation of the mTOR downstream factors S6 and S6K1 [28], [29]. The phosphorylation status of S6 and S6K1 can provide an index of mTOR activity [30]. In leucine experiment III, cells expressing co-localized c-Fos and pS6 in food related brain areas were quantified, to determine the number of activated neurons in the mTOR pathway after either leucine or valine injections in *Slc6a15* KO and WT mice. One outlier was found with Grubbs' test (GraphPad, USA), and excluded from the data set before statistical analysis. Leucine injections non-significantly increased the number of c-Fos/pS6 co-positive cells in VMH (p = 0.1581) and in PVN (p = 0.3740) in WT compared to *Slc6a15* KO mice. WT mice had a non-significantly lower number of cells co-labelled with c-Fos and pS6 in Arc (p = 0.2264) and in DMH (p^Leu^ = 0.1391) in WT compared to *Slc6a15* KO mice (Fig. 2F). In conclusion, no differences were seen between the two groups in any of the studied brain regions after leucine injections. Valine induced significant reduction of activated neurons in the mTOR pathway in VMH (p = 0.0096) in WT compared to *Slc6a15* KO mice, and gave non-significant increased activation in PVN (p = 0.1542) and in DMH (p = 0.1889) in WT compared to *Slc6a15* KO mice. No differences between the two groups were seen in Arc (p = 0.9102) after valine injections (Fig. 2G). In conclusion, a significant difference between the two groups was only seen in VMH after valine injections. When quantifying cells in VMH, DMH and in Arc, the double c-Fos and pS6 labeling extended over a wider rostro/caudal extent than c-Fos labeling alone. The expression pattern was still similar to the single labeling after leucine injection, while valine injection only gave a similar pattern in DMH. The counted cells with c-Fos staining co-localizing with pS6 staining in leucine experiment III followed the pattern of the c-Fos data in leucine experiment II for most of the brain regions. Figure S4A illustrates the c-Fos staining with activated neurons visualized with black labelled nucleus, while figure S4B illustrates the double staining with c-Fos labelling the nucleus in black and pS6 labelling the whole cell body in brown.

### Leucine supplemented mice show down-regulation of Slc6a15, Slc6a17, Mtor, Rsp6 and Eif4e in PVN

**Figure 3:**
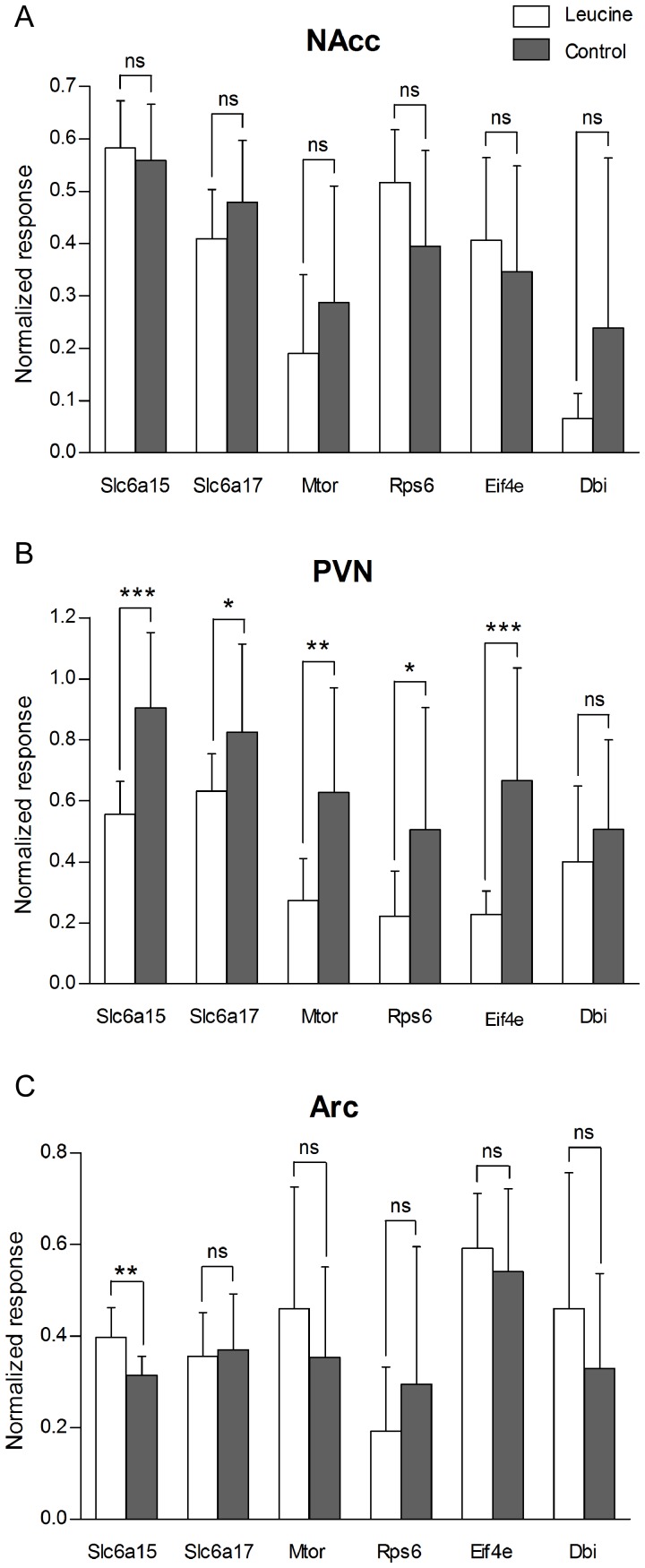
Down-regulation of mRNA expression for *Slc6a15, Slc6a17*, *Mtor* and downstream factors in the *mTOR* pathway in the PVN in leucine supplemented WT mice. Effect of leucine on mRNA expression of *Slc6a15* and *Slc6a17*, *Mtor* and the *Mtor* downstream factors *Rps6* and *Eif4e*, and *Dbi*. Real-time PCR data run on cDNA from the nucleus accumbens (Nacc) (A), paraventricular nucleus (PVN) (B), and arcuate hypothalamic nucleus (Arc) (C), prepared from mice given leucine for 48 h and with mice given regular water as a control group (n = 10–12/group). (A) No difference in mRNA expression in NAcc was detected between the two groups. (B) Leucine-fed mice expressed significantly lower levels of *Slc6a15* (p = 0.0002), *Slc6a17* (p = 0.0449), *Mtor* (p = 0.0044), *Rps6* (p = 0.0309) and *Eif4e* (p = 0.0006) mRNA in PVN than mice given water. *Dbi* showed a similar trend (p = 0.3713). (C) Leucine-fed mice had down-regulation of *Slc6a15* mRNA in Arc (p = 0.0015), whereas levels of *Slc6a17*, *Mtor*, *Rps6*, *Eif4e*, and *Dbi* mRNA were unaffected. Asterisks denote significance of Student's t-test for difference of means (*p<0.05, **p<0.01, ***p<0.001).

Real-time PCR was used to determine the relative changes in expression of genes involved in leucine transport and mTOR signalling in WT mice fed leucine. Gene expression was investigated in NAcc, PVN and Arc after 48 hours of leucine exposure, see Fig. 3. Neither the *Slc6a15* transporter, the closely related *Slc6a17* [8], *Mtor* or the downstream factors *Rps6* or *Eif4e* significantly changed expression in the NAcc of leucine-fed mice (Fig. 3A). However, in PVN, the leucine-fed mice expressed significantly lower levels of *Slc6a15* (p = 0.0002), *Slc6a17* (p = 0.0449), *Mtor* (p = 0.0044), *Rps6* (p = 0.0309) and *Eif4e* (p = 0.0006) mRNAs (Fig. 3B). The mRNA for the peptide *Dbi* (p = 0.3713) showed no difference in expression. The *Slc6a15* transporter mRNA was significantly up-regulated in the Arc (p = 0.0015) in leucine-fed mice compared to the water control group, while mRNA expression of the other genes studied was not significantly affected (Fig. 3C).

### High Slc6a15 gene expression in CNS

**Figure 4:**
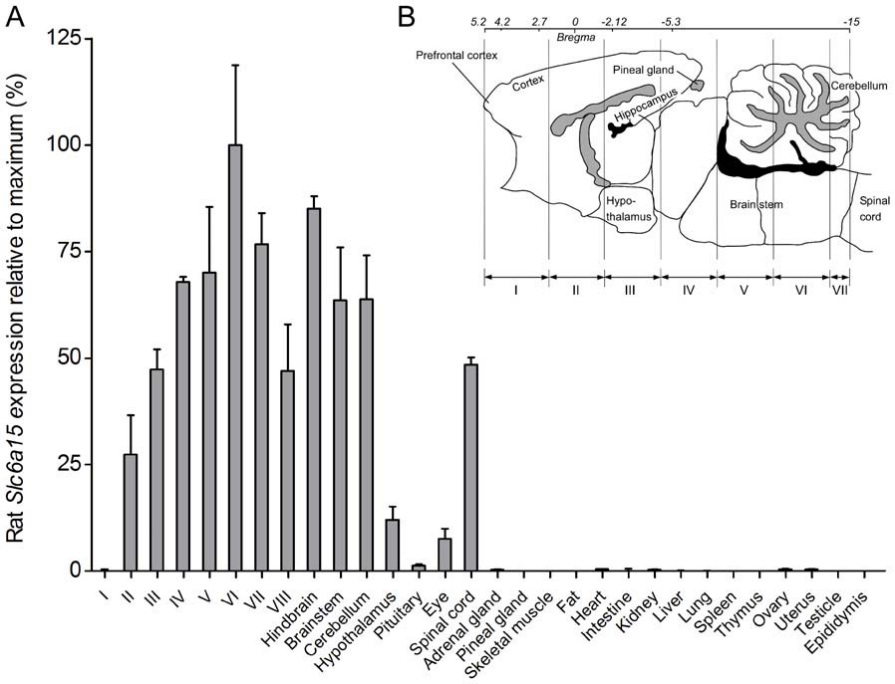
High mRNA expression of *Slc6a15* in CNS. Real-time PCR data visualized as column charts with standard deviations displaying the rat *Slc6a15* expression (% ± SD%) relative to maximum (fold decrease) in rat tissues. (**A**) *Slc6a15* show high mRNA expression in the brain and spinal cord and low or almost no expression in the peripheral tissues. The abbreviations I–VIII indicates the eight brain cross sections. (**B**) Schematic brain illustration of cross sections labelled I-VIII (redrawn from [18]).

Expression analysis of *Slc6a15* was performed in rat CNS and peripheral tissues, see Fig. 4. *Slc6a15* mRNA had widespread, multifocal expression in the CNS and low or almost no expression in the peripheral tissues (Fig. 4A). Relative expression below 1% was seen in adrenal gland, kidney, ovary and uterus, and very low or absent expression noted in brain slice I, pineal gland, skeletal muscle, fat, intestine, liver, lung, spleen, thymus, testicle and epididymis. A schematic illustration of the dissection of the eight brain sections (I–VIII) is shown in Fig. 4B.

### Slc6a15 has abundant mRNA expression in the brain

**Figure 5:**
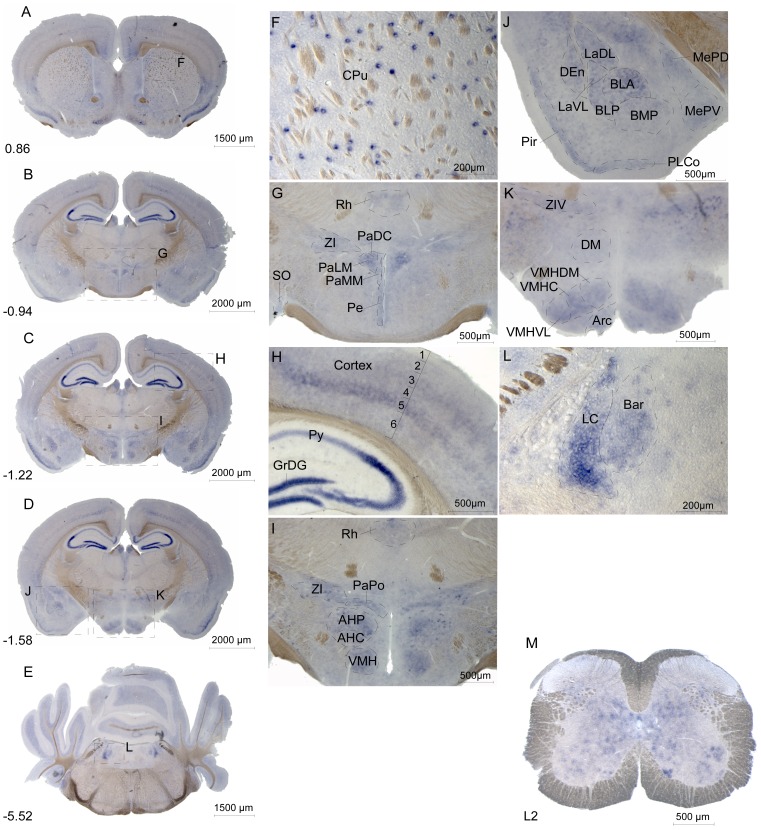
*Slc6a15* mRNA expression in mouse brain and spinal cord. Floating *in situ* hybridization using 500 ng digoxigenin labelled mouse *Slc6a15* probe to detect cells and nuclei populations expressing the mRNA, with overview image of coronal mouse brain sections (**A–E**), close up images (**F–L**) and spinal cord (**M**). The Bregma coordinates, abbreviations and described brain regions is depicted using Franklin and Paxinos 2007 [18]. *Slc6a15* mRNA brain expression is found in following brain regions. (**F**) Patches of caudate putamen (striatum, CPu). (**G**) Supraoptic nucleus (SO), rhomboid thalamic nucleus (Rh), zona incerta (ZI), paraventricular hypothalamic nucleus dorsal cap (PaDC), paraventricular hypothalamic nucleus lateral magnocellular part (PaLM), paraventricular hypothalamic nucleus medial mag nocellular part (PaMM) and periventricular hypothalamic nucleus (Pe). (**H**) Cortical layer 2–6, granule cell layer of the dentate gyrus (GrDG) and pyramidal cell layer of the hippocampus (Py). (**I**) Rhomboid thalamic nucleus (Rh), zona incerta (ZI), paraventricular hypothalamic nucleus posterior part (PaPo), anterior hypothalamic area, posterior part (AHP), anterior hypothalamic area, central part (AHC) and ventromedial hypothalamic nucleus (VMH). (**J**) Lateral amygdaloid nucleus dorsolateral part (LaDL), lateral amygdaloid nucleus ventrolateral part (LaVL), dorsal endopiriform claustrum (DEn), basolateral amygdaloid nucleus anterior part (BLA), basolateral amygdaloid nucleus posterior part (BLP), basomedial amygdaloid nucleus posterior part (BMP), piriform cortex (Pir), posterolateral cortical amygdaloid area (PLCo), medial amygdaloid nucleus posterodorsal part (MePD) and medial amygdaloid nucleus posteroventral part (MePV). (**K**) Zona incerta ventral part (ZIV), dorsomedial hypothalamic nucleus (DM), ventromedial hypothalamic nucleus dorsomedial part (VMHDM), ventromedial hypothalamic nucleus central part (VMHC), ventromedial hypothalamic nucleus ventrolateral part (VMHVL) and arcuate hypothalamic nucleus (Arc). (**L**) Locus coeruleus (LC) and Barrington's nucleus (Bar). (**M**) The spinal cord mRNA expression of *Slc6a15* is found in subsets of somatic motor neurons and interneurons in the upper vertebrae L2 lumbar.

We used *in situ* hybridization to evaluate *Slc6a15* mRNA expression in adult male mouse brain and spinal cord. Expression of *Slc6a15* was high in striatum, cortex, hippocampus, amygdala, hypothalamus and pons, (Fig. 5 and Table S3) and in spinal cord (Fig. 5M). Interestingly, high expression was found in CPu, especially in the striosome-like patches (Fig. 5A and F). There was expression surrounding the third ventricle in periventricular hypothalamic nucleus (Fig. 5B and G). Other striatal nuclei with staining are listed in Table S3. The *Slc6a15* gene was highly expressed in cortical layer 5, known to contain many glutamatergic neurons [31], while expression was lower in the other layers and absent in layer 1 (Fig. 5C and H). Further, *Slc6a15* mRNA was detected in the Pir (Fig. 5D and J). In the hippocampus, *Slc6a15* mRNA was found to be strongly expressed in the GrDG and in neurons of the pyramidal cell layer (Fig. 5C and H). Strong expression of the *Slc6a15* gene was found in the SON (Fig. 5B and G), VMH (Fig. 5D and K), AH (Fig. 5C and I), BLA (Fig. 5D and J), Arc (Fig. 5D and K) and in pons in LC (Fig. 5E and L). These are areas known to be part of the regulation of food intake [32], [33]. In contrast, only low mRNA expression was found in thalamus and cerebellum. Widespread *Slc6a15* mRNA expression was detected in the spinal cord, where the gene was detected in subsets of somatic motor neurons and in interneurons (Fig. 5M).

### Cellular localization of B^0^AT2 in neurons, astrocytes and DBI positive cells

**Figure 6:**
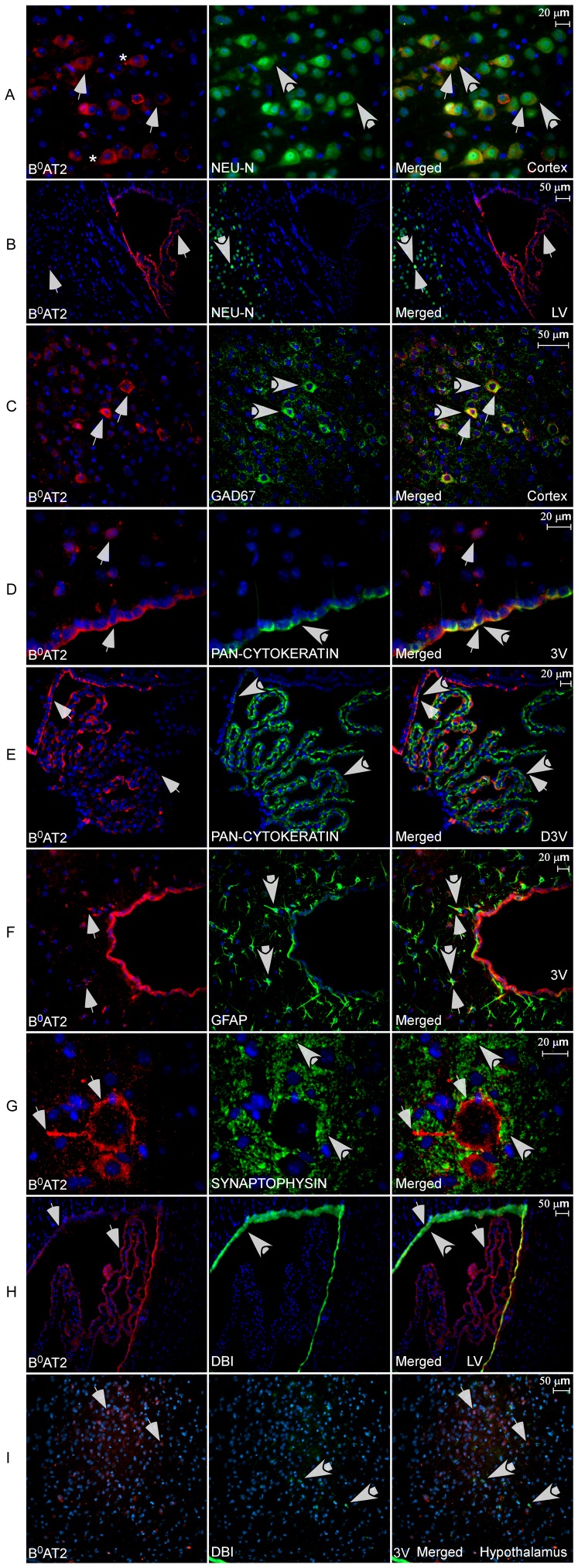
Cellular localization of the B^0^AT2 protein in mouse brain and spinal cord. Immunohistochemistry on free floating adult mouse brain sections using the polyclonal B^0^AT2 antibody (red), cell nucleus marker DAPI (blue), and antibody markers (green). Thin arrows indicate B^0^AT2 expressing cells and thick arrows cells labelled with markers. (**A**) The B^0^AT2 antibody stained cells in the cell membrane and in the axons, asterisk indicate a labelled axon. The B^0^AT2 protein and neuronal marker NeuN had similar expression patterns in cortex and a number of cells showed co-localization. (**B**) B^0^AT2 staining in cells surrounding and within the lateral ventricle (LV) as well as in a few cells in the inner layer of cortex. Co-localization of B^0^AT2 and NeuN was shown only in a few cells in the cortex. (**C**) High overlap of B^0^AT2 and GABAergic neuronal marker GAD67 in cortex. (**D**) B^0^AT2 protein localized to the cytoplasm around the nuclei in cells surrounding the third ventricle (3V) and in other hypothalamic cells, and high overlap was shown between B^0^AT2 and the epithelial marker pan-Cytokeratin. (**E**) High overlap between the B^0^AT2 protein and pan-Cytokeratin within the dorsal third ventricle (D3V). (**F**) B^0^AT2 showed expression in both cells surrounding the 3V and other hypothalamic cells, while the astrocyte marker GFAP expressing cells were located more close to the 3V. A number of B^0^AT2 expressing cells co-localized with GFAP. (**G**) B^0^AT2 expression in motor neurons in spinal cord (L2), with B^0^AT2 labelling the cytosol and the neuronal axons. B^0^AT2-labelled cells did not co-localize with the vesicle marker synaptophysin. (**H**) B^0^AT2 was found in cells following the edge of the LV and in cells within the ventricle, while the DBI positive cells was only found in ependymocytes bordering the ventricle. High overlap was seen for B^0^AT2 and the product of the DBI gene. (**I**) B^0^AT2 expression in hypothalamus, with a number of cells stained close to the upper part of the 3V. The DBI positive cells were located in the epithelial cells surrounding the ventricle and spread though the hypothalamus. Co-localization of B^0^AT2 and DBI positive cells was only found in the epithelial cells. The A, C, D and G images are photographed with 40× magnification and 20× magnification was used for the B, E, F, H and I images. Row A and B are at Bregma −0.46, row C–E at −1.58 and row F, H and I at −0.82.

**Figure 7:**
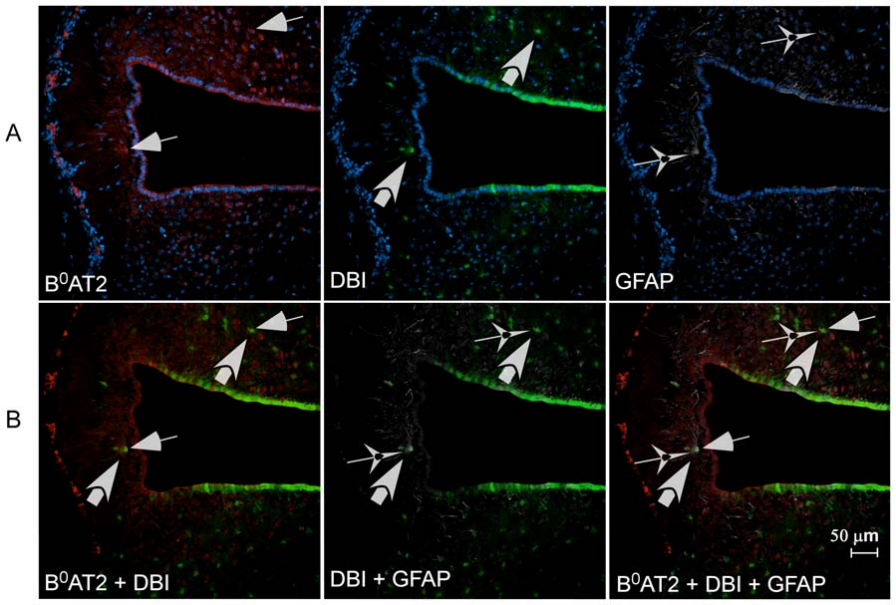
Cellular co-localization of B^0^AT2, GFAP and DBI positive cells. Immunohistochemistry on free floating adult mouse brain sections using the polyclonal B^0^AT2 antibody (red), cell nucleus marker DAPI (blue), DBI marker (green), and GFAP marker (white). Thin arrows indicate B^0^AT2 expressing cells, thick arrows DBI labelled cells and star-like arrows GFAP expressing cells. **A row**; Hypothalamic expression of B^0^AT2 both close to the third ventricle and in the surrounding areas. The expression of the two markers DBI and GFAP is seen in cells surrounding the ventricle and the border of the brain section. **B row**; Co-localization of B^0^AT2 and GFAP and the co-localization of B^0^AT2 and DBI expressing cells only in cells close to the ventricle and in epithelial cells surrounding the third ventricle. The section is at Bregma −1.06 and 20× magnification was used for the image.

We generated a rabbit polyclonal B^0^AT2 antibody. Western blot analysis documented the specificity of the antibody (Fig. S2). We used immunohistochemistry with this B^0^AT2 antibody and a number of antibody markers to identify the cell types expressing B^0^AT2, shown in Fig. 6. The co-localization of B^0^AT2 and the neuron-specific DNA-binding protein marker (NeuN) [34] in the cortex suggested neuronal expression of the B^0^AT2 protein in the brain. B^0^AT2 was localized to the soma and the neuronal axons (Fig. 6A and B). All cells stained with NeuN were not stained with B^0^AT2, indicating that the B^0^AT2 protein is neuronally expressed, but not in all neurons. The B^0^AT2 protein was further localized to GABAergic neurons (Fig. 6C), where the glutamic acid decarboxylase 67 protein (GAD67) [35] marker was used for co-localization. All cells labelled with B^0^AT2 did not express the GAD67 protein, suggesting expression in both inhibitory and non-inhibitory neurons. Additional co-localization with B^0^AT2 and the neuron-specific antibody markers pan-neuronal, NSE and MAP2, showed overlapping expression in cerebral cortex and in dorsal third ventricle (D3V) (Fig. S3A-E). B^0^AT2 immunoreactivity co-localizes with the choroid plexus epithelial cell cytokeratin marker [36] in cells surrounding the third ventricle (3V) (Fig. 6D) as well as other ventricles and in cells within the D3V (Fig. 6E), showing that the B^0^AT2 protein is expressed in choroid plexus epithelial cells. Interestingly, the B^0^AT2 transporter showed high expression in a limited number of cells surrounding the ventricles in brain, which all co-localize with the astrocyte marker glial fibrillary acidic protein (GFAP) [37]. The overlap between GFAP and B^0^AT2 protein was only seen close to the ventricles (Fig. 6F). The B^0^AT2 protein did, however, not co-localize with presynaptic vesicle protein synaptophysin in the spinal cord [38] (Fig. 6G). The spinal cord expression showed localization of the B^0^AT2 protein within large motor neurons, with high protein expression localized to the cytoplasm, in the cell membrane, and in the neuronal axons. Finally, co-localization of the B^0^AT2 protein and the protein product of the *Dbi* gene, previously shown to be localized in glial cells [39], [40], were seen around the lateral ventricle (LV) and hypothalamus (Fig. 6H and I). The immunohistochemistry showed co-expression of B^0^AT2 protein and DBI in cells following the edge of the LV, suggesting B^0^AT2 expression in ependymocytes bordering the ventricles in the brain. All cells expressing DBI did not express the B^0^AT2 protein. Co-localization of the B^0^AT2 protein, the astrocyte marker GFAP and DBI positive cells was only found close to the ventricles and in the cell layer surrounding the ventricles, as shown in Figure 7. Taken together, the data indicated B^0^AT2 expression not only in neurons, but also in astrocytes and enriched in epithelial cells surrounding the ventricles in the brain. There is the caution, however, that many antisera provide nonspecific staining of choroid plexus and ependyma.

### B^0^AT2 expression in WT mice vs. SLC6A15 KO mice

**Figure 8:**
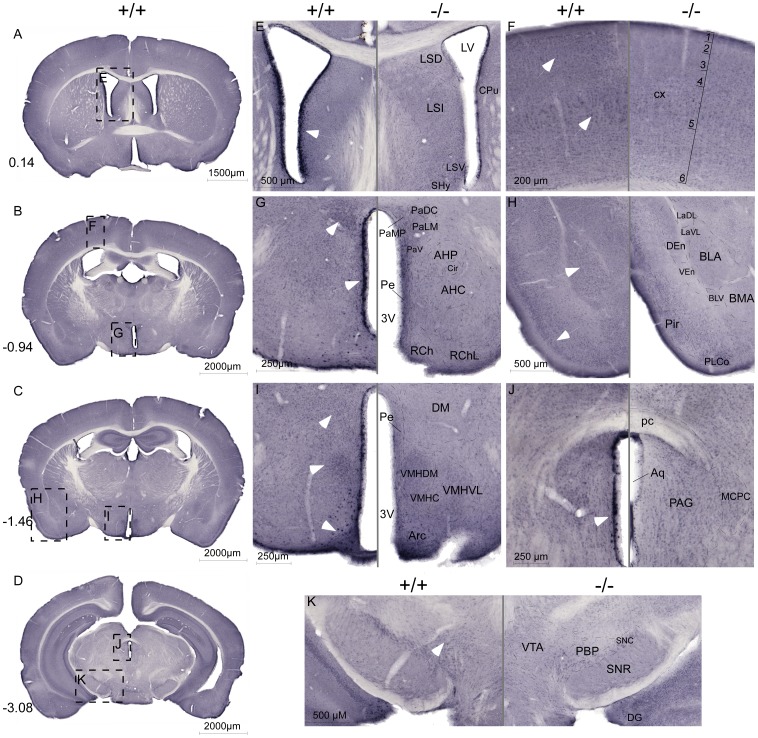
High B^0^AT2 expression in hypothalamus and in cells surrounding ventricles in WT mice. Non-fluorescent immunohistochemistry on free floating brain sections from WT (+/+) mice and *Slc6a15* KO (−/−) mice using the B^0^AT2 antibody. Overview images (**A–D**) of sections from WT mice with Bregma level in the left corner. Close up images (**E–K**) with WT sections on the left panel, and with the corresponding *Slc6a15* KO section with annotations on the right panel. White arrows indicate high B^0^AT2 expression in WT mice. (**E**) High B^0^AT2 expression in cells surrounding lateral ventricle in WT mice. (**F**) Stronger B^0^AT2 expression in layer 3 and 5 in cortex in WT mice than in *Slc6a15* KO mice. (**G**) Strong hypothalamic expression of B^0^AT2 in PVN and in Pe in WT mice. (**H**) High levels of B^0^AT2 in BLA in amygdala and in Pir in WT mice. (**I**) High hypothalamic B^0^AT2 staining in WT mice in DM, VMH and in Arc. (**J**) High expression of B^0^AT2 in cells surrounding the aqueduct in WT mice. (K) B^0^AT2 staining in the VTA in WT mice. Abbreviations: lateral septal nucleus, dorsal part (LSD), lateral ventricle (LV), caudate putamen (striatum) (CPu), lateral septal nucleus, intermediate part (LSI), lateral septal nucleus, ventral part (LSV), septohypothalamic nucleus (SHy), cortex (cx), paraventricular hypothalamic nucleus, dorsal cap (PaDC), paraventricular hypothalamic nucleus, lateral magnoce (PaLM), paraventricular hypothalamic nucleus, medial parvicel (PaMP), paraventricular hypothalamic nucleus, ventral part (PaV), anterior hypothalamic area, posterior part (AHP), circular nucleus (Cir), anterior hypothalamic area, central part (AHC), periventricular hypothalamic nucleus (Pe), 3 rd ventricle (3 V), retrochiasmatic area (RCh), retrochiasmatic area, lateral part (RChL), lateral amygdaloid nucleus, dorsolateral part (LaDL), lateral amygdaloid nucleus, ventrolateral part (LaVL), dorsal endopiriform claustrum (DEn), ventral endopiriform claustrum (VEn), basolateral amygdaloid nucleus, anterior part (BLA), basolateral amygdaloid nucleus, ventral part (BLV), basomedial amygdaloid nucleus, anterior part (BMA), posterolateral cortical amygdaloid area (PLCo), piriform cortex (Pir), dorsomedial hypothalamic nucleus (DM), ventromedial hypothalamic nucleus, dorsomedial part (VMHDM), ventromedial hypothalamic nucleus, central part (VMHC), ventromedial hypothalamic nucleus, ventrolateral part (VMHVL), arcuate hypothalamic nucleus (Arc), posterior commissure (pc), aqueduct (Aq), periaqueductal gray (PAG), magnocellular nucleus of the posterior commissure (MCPC), ventral tegmental area (VTA), parabrachial pigmented nucleus of the VTA (PBP), substantia nigra, compact part (SNC), substantia nigra, reticular part (SNR) and dentate gyrus (DG). Abbreviations and described brain regions were depicted using Franklin and Paxinos 2007 [18].

The protein expression of the B^0^AT2 transporter and the specificity of the B^0^AT2 antibody was investigated using immunohistochemistry and the polyclonal B^0^AT2 antibody, shown in Figure 8. The B^0^AT2 antibody gave strong labelling of cells on sections from the WT mice, especially in cells surrounding the ventricles, in layers of cortex and in hypothalamus, a staining that was missing or very weak in sections from the *Slc6a15* KO mice. The B^0^AT2 expression was high in WT mice in cells surrounding lateral ventricle (Fig. 8A and E), in cells close to the third ventricle (Fig. 8B and G), and close to the aqueduct (Fig. 8D and J). The *Slc6a15* KO mice lacked the strong characteristic B^0^AT2 expression in cell layer 3 and 5 in cerebral cortex (Fig. 8B and F) and in Pir (Fig. 8C and H), a staining clearly visualized in the WT mice. The transporter showed strong hypothalamic expression in PVN (Fig. 8B and G), and in DM, VMH and Arc in WT mice (Fig. 8C and I). B^0^AT2 also showed strong staining in BLA (Fig. 8C and H) and in the VTA (Fig. 8D and K) in WT mice, an expression pattern that together with the hypothalamic expression gives strength to the hypothesis that the B^0^AT2 transporter could be involved in the regulation of food intake.

## Discussion and Conclusions

The overall uptake of leucine into the CNS is greater than for any other amino acid [41] and therefore leucine has been proposed to play a critical role both as a major nitrogen precursor for the cerebral glutamate, GABA and glutamine synthesis and as a nitrogen carrier within the brain [42]–[44]. We show here that B^0^AT2 is expressed in GABAergic neurons as well as in regions rich in glutamatergic neurons, suggesting that the transporter plays a role in these processes. It has also been shown that leucine mediates anorexigenic effects when given to animals and thus plays a role in regulating food intake [25]. In this study we show that the amino acid transporter B^0^AT2 (the product of the *Slc6a15* gene) is important for mediating the anorexigenic response to rapid administration of leucine by utilizing the *Slc6a15* KO mice [11]. Our expression microarray analysis showed no clustering by genotype. Also, expression of none of the genes on the array were significantly up- or down-regulated in *Slc6a15* KO mice compared to WT when adjusted for multiple testing using FDR [23], and pathway analysis showed that the genes with the most differing? expression levels between the groups were involved in basic cellular functions (Table 1). This suggests that there could be compensatory mechanisms acting in the KO mice to affect basic organization of the brain and that there are no major compensatory mechanisms acting on the regulatory level of receptors and other transporters. This indicates that the phenotype of the *Slc6a15* KO mice may not be revealed unless the system is challenged pharmacologically or otherwise. The results from the expression microarray were in good agreement with the results of the initial characterization of the *Slc6a15* KO mice, where no obvious phenotypes were detected in basal comparisons with WT mice [11].

It has previously been shown that B^0^AT2 is a high affinity transporter of leucine [9]. Of the 56 known amino acid transporters there are only two, SLC6A15 and SLC6A17, with high affinity for leucine expressed in brain [45]. The neuronally expressed SLC7 family members SLC7A5, SLC7A8, SLC7A9, and SLC7A10, also transport leucine but with modest affinity [46]–[49]. Because the SLC6A17 protein has been suggested to be expressed on vesicles [50]–[53], B^0^AT2 may represent the best candidate among the known transporters to mediate the anorexigenic response to leucine. In this study we measured food intake after leucine or valine injections in *Slc6a15* KO and WT mice. Valine, also a substrate for B^0^AT2 transport [9], may not affect feeding as robustly as leucine in WT mice [5], [25]. We tested our hypothesis that reduced food intake and different neuronal activation in food related brain areas should be seen in response to leucine and valine injections in *Slc6a15* KO mice compared to WT. We chose to use valine with similar caloric content as control, as it has been previously used [5], [25]. Intraperitoneal injections avoid activation of response systems in the intestines, such as ghrelin, which can induce activation of neurons in food related areas in the brain [54]. Also, these injections avoid the taste pathways for amino acids. We showed a significant reduction in food intake in WT mice compared to *Slc6a15* KO after leucine injections, but not after valine injections, showing that B^0^AT2 plays a role in mediating the anorexigenic effects of leucine (Fig. 2B). B^0^AT2 expression in the epithelial cells around the ventricles (Fig. 6D, E and H) and its dense neuronal expression close to the ventricles and in key hypothalamic areas (Fig. 8G and I) provide multiple sites at which B^0^AT2-mediated uptake of leucine could relay information could relay information about systemic and or CSF leucine concentrations to neurons.

Blouet *et al.* showed that leucine injection activates neurons in food related brain areas, such as Arc, PVN and NTS, as assessed by increased c-Fos expression [25]. We showed that leucine injection not only reduced food intake, but also induced c-Fos activation in the VMH in WT mice with attenuated response in *Slc6a15* KO (Fig. 2D). VMH is a known satiety centre and lesions of either VMH or PVN lead to hyperphagia and obesity [55]. Our results suggest that B^0^AT2 is an essential component of leucine activated signalling in hypothalamic neurons related to food intake and satiety. After leucine injections, our data also showed a trend toward decreased c-Fos activation in Arc and in DMH in WT mice compared to *Slc6a15* KO. The satiety pathways in Arc can be activated by ingestion of proteins, resulting in activation of POMC neurons and α-MSH neurons, and a down-regulation of NPY neurons. Intake of proteins leads to increased plasma concentrations of amino acids, and can result in activation of Arc satiety pathways ending up in reduced food intake [56], [57]. DMH is known to integrate and process information from the Arc and the VMH and has a role in the modulation of energy intake. Similar to the effect of lesions in VMH, lesions in the DMH result in hyperphagia and obesity, but the effect size is much smaller [58], [59]. All these regions have been implicated in the control of energy homeostasis [60]. Injection(s) of leucine did not induce a significant increase in the number of c-Fos positive neurons in PVN. The number of activated neurons in the mTOR pathway after leucine injections showed similar, but non-significant trends, as for the c-Fos data (Fig. 2F). Leucine can be sensed by mTOR, which can lead to activation of POMC neurons [5]. It would therefore be of interest to further study the activation of POMC neurons and down-regulation of NPY after leucine injections in *Slc6a15* KO and WT mice, to investigate the effects of B^0^AT2 to mediate satiety. Our results strongly support B^0^AT2 as part of leucine activated signalling in hypothalamic neurons related to food intake.

The RT-PCR data are interesting as we show altered gene expression in the hypothalamic areas PVN and Arc of leucine-fed mice with reduced expression of genes in the mTOR pathway in PVN (Fig. 3). A previous study has investigated the effects on the mTOR pathway at protein level in other areas such as hypothalamus, cerebral cortex and hippocampus, showing only effects in Arc in hypothalamus [5]. The down regulation we observe here is possibly a negative feedback response to exposure of increased leucine levels. This suggests that prolonged exposure to amino acids would reduce the sensitivity of this satiety system, which has implications of usage of leucine as an anti-obesity agent.

The B^0^AT2 transporter is known to be expressed in the adult and fetal brain [7], [9], [12]. We performed a thorough expression analysis in mouse brain using *in situ* hybridization (Fig. 5). We found high expression of the *Slc6a15* gene in hypothalamic areas such as SON, VMH, AH, PVN, and in Arc. We also saw *Slc6a15* mRNA expression in amygdala, Pir and LC. These results confirm and extend those of other *in situ* hybridization studies of *Slc6a15* [13]–[15], [19]. In correlation with our *in situ* hybridization results, B^0^AT2 immunoreactivity was seen mainly in neurons, localized to GABAergic as well as other neurons. In general, all cells expressing the neuronal markers did not express B^0^AT2, indicating that the B^0^AT2 protein is neuronally expressed in both inhibitory and non-inhibitory neurons, but not in all neurons (Fig. 6A–C). This expression pattern and the cellular localization strengthens the possibility that SLC6A15 takes part in various aspects of a amino acid sensor system [61], [62], in food intake regulation [32], [33] and functions as a provider of neurotransmitter precursors in many neurons.

Interestingly, we saw B^0^AT2 immunoreactivity in some astrocytes close to the third ventricle and in choroid plexus, a structure in the ventricles of the brain surrounded by cerebrospinal fluid (Fig. 6F–G). B^0^AT2 has never previously been localized to astrocytes, suggesting B^0^AT2 to contribute to the uptake of circulating amino acids from the bloodstream into the brain. We did find that B^0^AT2 co-localizes with the diazepam binding inhibitor (DBI) [63], in astrocytes close to the ventricles and in ependymocytes bordering the ventricles (Fig. 6H and Fig. 7). DBI is expressed in hypothalamus and circumventricular organs, especially in astroglial and ependymal cells [39], [40], and is believed to play a significant role in intracellular acyl-CoA transfer and pool formation [64]. The co-localization of B^0^AT2 to cells expressing DBI is in good agreement with our hypothesis that B^0^AT2 might play an important role in regulation of brain levels of leucine and other BCAAs.

Taken together, this work provides evidence for B^0^AT2 to be involved in neuronal responses to changes in leucine concentrations, with leucine concentrations being a measure of the energy status of the organism and hence a direct way to regulate energy balance through nutrients.

## Supporting Information

Figure S1 **Vector map of the mmSlc6a15 clone used for producing the** ***in situ*** **hybridization probe.** The restriction enzyme XmnI and the T3 RNA polymerase were used to synthesize the DIG-labelled anti-sense mRNA probe (602 bp) containing the last coding exon in the gene. Restriction enzymes occurring one time in the vector were labelled in red, if occurring two times labelled in green.
(TIF)

Figure S2 **Characterization of the B^0^AT2 antibody.** Western blot was performed to investigate the specificity of the custom made polyclonal B^0^AT2 antibody. Crude lysate A (50 µg) and B (200 µg) from WT mouse brain was loaded to the gel and transferred to a membrane with ladder (kDa) loaded on the left edge. The primary B^0^AT2 antibody was added for binding after pre-blocking. A specific strong band was seen at ∼ 85 kDa (upper red arrow) and a weak band at ∼ 45 kDa (lower red arrow). The epitope for the antibody is in the N-termini of the protein and the actual size of the B^0^AT2 protein is 81.9 kDa, with one isoform containing the N-termini known to be 29.9 kDa (NM_182767 and NM_018057). The bands acquired indicate binding of the antibody to both the actual B^0^AT2 protein and to the 29.9 kDa isoform, both containing the N-termini of the protein, and thereby shows that the custom made polyclonal B^0^AT2 antibody was epitope specific.
(TIF)

Figure S3 **Neuronal expression of the B^0^AT2 protein.** Immunohistochemistry on free floating adult mouse spinal cord sections using the polyclonal B^0^AT2 antibody in red and the cell nucleus marker DAPI in blue. All the markers, neuron-specific pan-neuronal antibody, the neuron specific enolase (NSE) antibody and the microtubule-associated protein 2 (MAP2), were stained in green. **A** and **B rows**; B^0^AT2 co-localization with the cocktail marker pan-neuronal in cortex (**A**) and dorsal third ventricle (D3V) (**B**), not all cells labelled with pan-neuronal were expressing B^0^AT2. **C** and **D rows**; High overlap of B^0^AT2 and NSE in cortical neurons (**C**) and D3V (**D**). **E row**; Both B^0^AT2 and MAP2 were expressed in the cortex and the merged picture showed extensive overlap between B^0^AT2 and MAP2. Fluorescent immunohistochemistry was performed on paraffin embedded mouse brain sections according to as described in the main text.
(TIF)

Figure S4 **(A) Immunohistochemical staining for c-Fos (black arrows).** (**B**) Double immunohistochemistry with c-Fos and pS6 antibodies gave black c-Fos staining (black arrows) in the nucleus and brown pS6 staining (gray arrows) in the whole cell, representing activated neurons in the mTOR pathway. Illustrations with abbreviations from Franklin and Paxinos 2007 [5]. Sections from a leucine injected SLC6A15 KO mouse. Bregma levels shown in the left corner. Immunohistochemistry was performed as described in the main text.
(TIF)

Table S1 **Antibody information.** Antibodies used for Western blot (WB), non-fluorescent (I) and fluorescent (FI) immunohistochemistry.
(DOCX)

Table S2 **Primer information.** Real-time PCR primers and reverse transcription PCR primers (all supplied Thermo Fisher Scientific, USA).
(DOCX)

Table S3 **Mouse brain expression of** ***Slc6a15*** **mRNA.** Scale of estimated expression; (+++) high expression, (++) medium expression, (+) low expression and (-) no apparent expression. The organization of the brain regions are described by using Franklin and Paxinos (2007) (Franklin and Paxinos, 2007).
(DOCX)
