# Supplementary figures and images for: B0AT2 (SLC6A15) Is Localized to Neurons and Astrocytes, and Is Involved in Mediating the Effect of Leucine in the Brain
Source: PLoS One. 2013 Mar 7;8(3):e58651. doi: 10.1371/journal.pone.0058651 (PMC3591439; doi:10.1371/journal.pone.0058651)

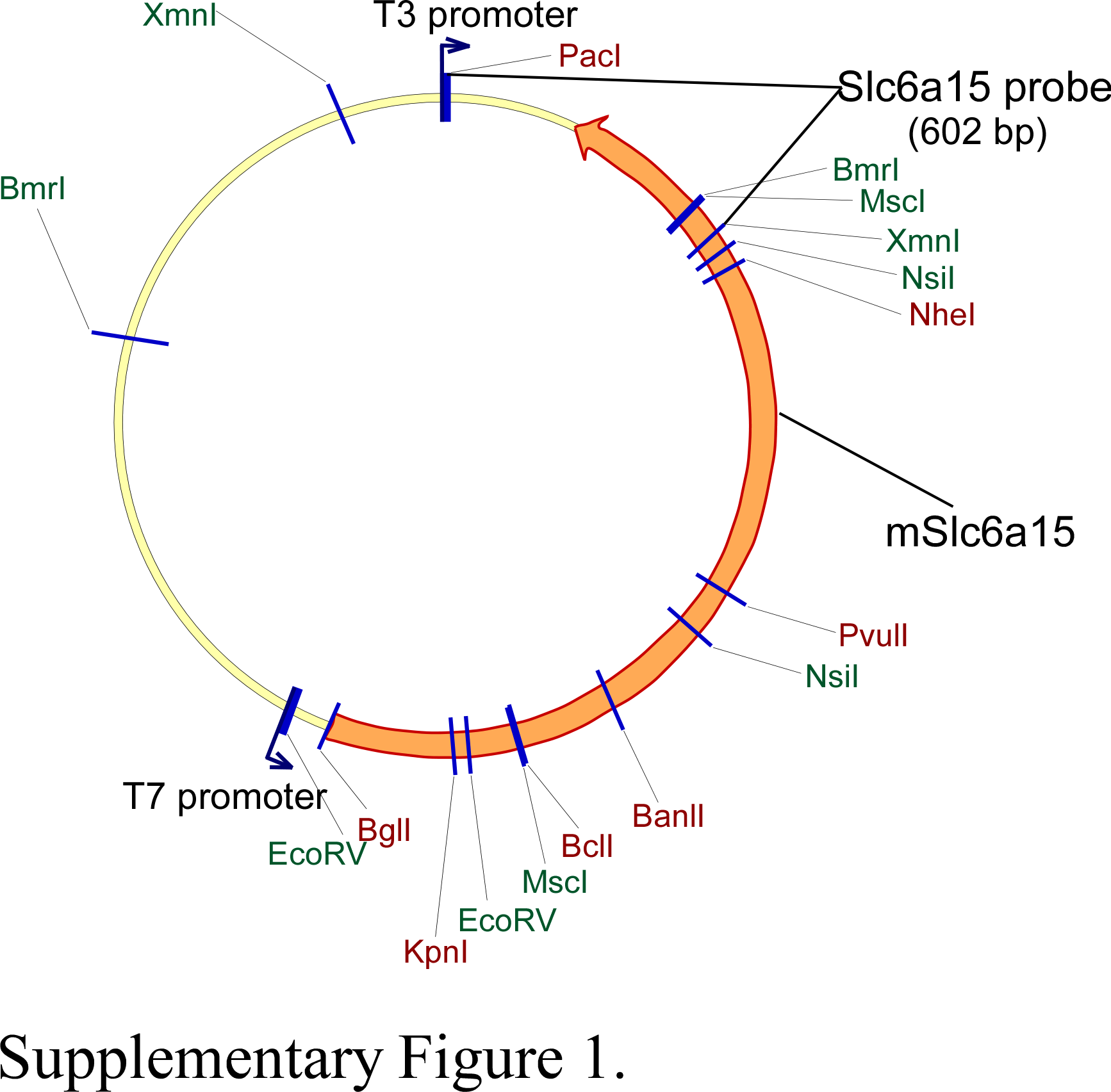

Supplement: Figure S1 — Vector map of the mmSlc6a15 clone used for producing the in situ hybridization probe. The restriction enzyme XmnI and the T3 RNA polymerase were used to synthesize the DIG-labelled anti-sense mRNA probe (602 bp) containing the last coding exon in the gene. Restriction enzymes occurring one time in the vector were labelled in red, if occurring two times labelled in green. (TIF) [file pone.0058651.s001.tif]

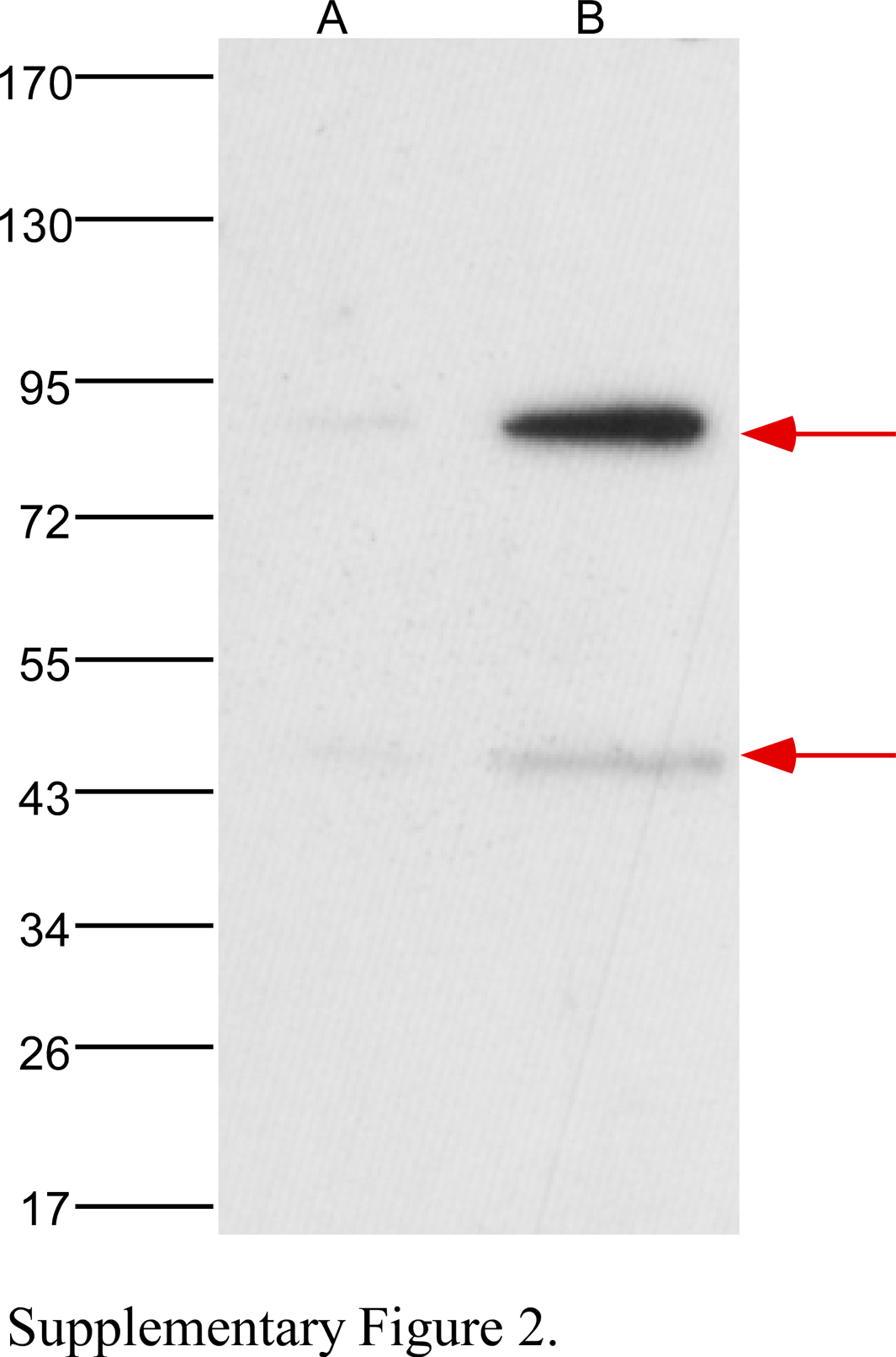

Supplement: Figure S2 — Characterization of the B0AT2 antibody. Western blot was performed to investigate the specificity of the custom made polyclonal B0AT2 antibody. Crude lysate A (50 µg) and B (200 µg) from WT mouse brain was loaded to the gel and transferred to a membrane with ladder (kDa) loaded on the left edge. The primary B0AT2 antibody was added for binding after pre-blocking. A specific strong band was seen at ∼ 85 kDa (upper red arrow) and a weak band at ∼ 45 kDa (lower red arrow). The epitope for the antibody is in the N-termini of the protein and the actual size of the B0AT2 protein is 81.9 kDa, with one isoform containing the N-termini known to be 29.9 kDa (NM_182767 and NM_018057). The bands acquired indicate binding of the antibody to both the actual B0AT2 protein and to the 29.9 kDa isoform, both containing the N-termini of the protein, and thereby shows that the custom made polyclonal B0AT2 antibody was epitope specific. (TIF) [file pone.0058651.s002.tif]

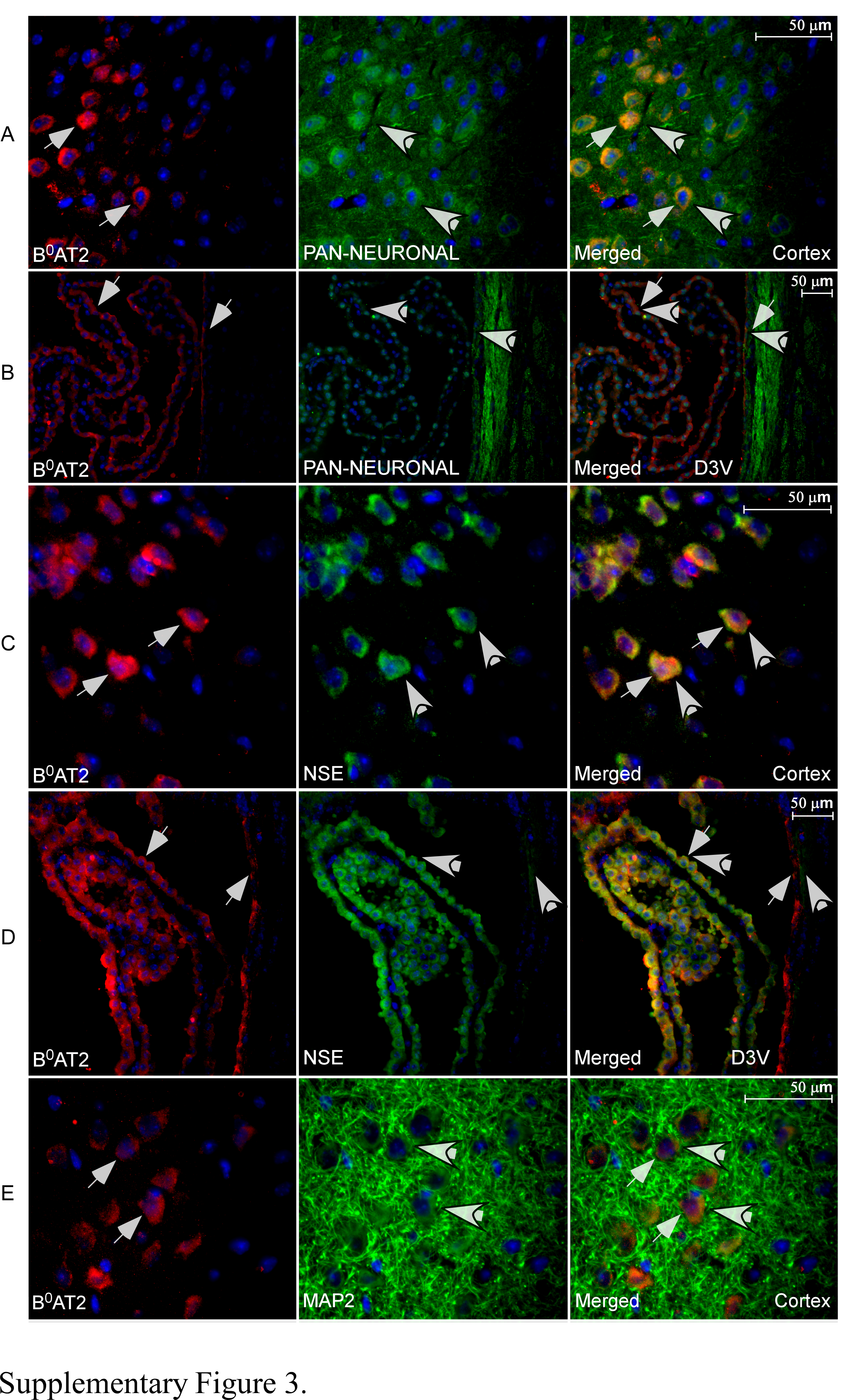

Supplement: Figure S3 — Neuronal expression of the B0AT2 protein. Immunohistochemistry on free floating adult mouse spinal cord sections using the polyclonal B0AT2 antibody in red and the cell nucleus marker DAPI in blue. All the markers, neuron-specific pan-neuronal antibody, the neuron specific enolase (NSE) antibody and the microtubule-associated protein 2 (MAP2), were stained in green. A and B rows; B0AT2 co-localization with the cocktail marker pan-neuronal in cortex (A) and dorsal third ventricle (D3V) (B), not all cells labelled with pan-neuronal were expressing B0AT2. C and D rows; High overlap of B0AT2 and NSE in cortical neurons (C) and D3V (D). E row; Both B0AT2 and MAP2 were expressed in the cortex and the merged picture showed extensive overlap between B0AT2 and MAP2. Fluorescent immunohistochemistry was performed on paraffin embedded mouse brain sections according to as described in the main text. (TIF) [file pone.0058651.s003.tif]

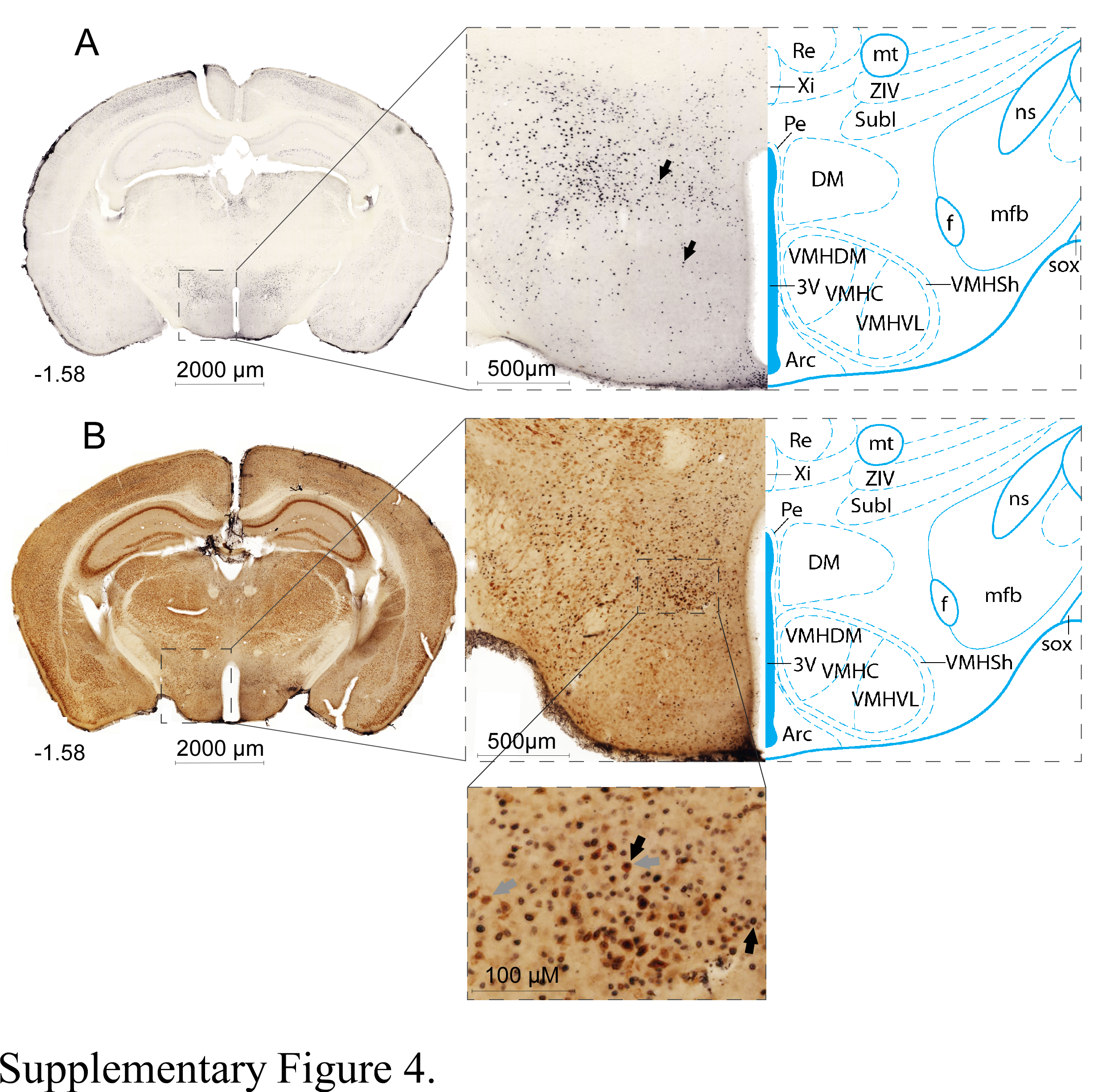

Supplement: Figure S4 — (A) Immunohistochemical staining for c-Fos (black arrows). (B) Double immunohistochemistry with c-Fos and pS6 antibodies gave black c-Fos staining (black arrows) in the nucleus and brown pS6 staining (gray arrows) in the whole cell, representing activated neurons in the mTOR pathway. Illustrations with abbreviations from Franklin and Paxinos 2007 [5]. Sections from a leucine injected SLC6A15 KO mouse. Bregma levels shown in the left corner. Immunohistochemistry was performed as described in the main text. (TIF) [file pone.0058651.s004.tif]
