# Supplementary material for: B0AT2 (SLC6A15) Is Localized to Neurons and Astrocytes, and Is Involved in Mediating the Effect of Leucine in the Brain
Source: PLoS One. 2013 Mar 7;8(3):e58651. doi: 10.1371/journal.pone.0058651 (PMC3591439; doi:10.1371/journal.pone.0058651)
Supplement: Table S1 — Antibody information. Antibodies used for Western blot (WB), non-fluorescent (I) and fluorescent (FI) immunohistochemistry. (DOCX) [file pone.0058651.s005.docx]

| **Primary antibodies** | **Species** | **Dilution** | **Company** |
| --- | --- | --- | --- |
| B^0^AT2 | Rabbit | 1:500 (WB)  1:5000 (I)  1:200 (FI) | Innovagen, Sweden |
| c-Fos | Rabbit | 1:2000 | Santa Cruz Biotechnology, USA |
| pS6 | Rabbit | 1:500 | Cell Signalling Technology, USA |
| NeuN | Mouse | 1:400 | Millipore, Sweden |
| Gad67 | Mouse | 1:200 | Millipore, Sweden |
| Pan-cytokeratin | Mouse | 1:200 | Sigma-Aldrich, USA |
| GFAP | Chicken | 1:400 | AbCam, United Kingdom |
| Synaptophysin | Mouse | 1:200 | BD Transduction lab, Sweden |
| DBI | Mouse | 1:100 | AbCam, United Kingdom |
| NSE | Chicken | 1:100 | AbCam, United Kingdom |
| Milli-mark pan-neuronal cocktail | Mouse | 1:100 | Millipore, Sweden |
| MAP2 | Mouse | 1:500 | Sigma-Aldrich, USA |
| **Secondary antibodies** |  | **Dilution** | **Company** |
| Anti-rabbit-horseradish peroxidase | Goat | 1:10000 | Invitrogen, USA |
| Anti-rabbit-biotin | Goat | 1:400 | Vector Laboratories, USA |
| Anti-rabbit-594 | Donkey | 1:200 | Invitrogen, USA |
| Anti-rabbit-594 | Goat | 1:200 | Invitrogen, USA |
| Anti-mouse-488 | Goat | 1:200 | Invitrogen, USA |
| Anti-mouse-488 | Chicken | 1:200 | Invitrogen, USA |
| Anti-chicken-647 | Goat | 1:200 | Invitrogen, USA |
| Anti-mouse-594 | Goat | 1:200 | Invitrogen, USA |
| Anti-chicken-488 | Goat | 1:200 | Invitrogen, USA |
