# Supplementary material for: B0AT2 (SLC6A15) Is Localized to Neurons and Astrocytes, and Is Involved in Mediating the Effect of Leucine in the Brain
Source: PLoS One. 2013 Mar 7;8(3):e58651. doi: 10.1371/journal.pone.0058651 (PMC3591439; doi:10.1371/journal.pone.0058651)
Supplement: Table S2 — Primer information. Real-time PCR primers and reverse transcription PCR primers (all supplied Thermo Fisher Scientific, USA). (DOCX) [file pone.0058651.s006.docx]

| **Gene** | **Accession nr. GenBank** | **Forward primer** | **Reverse primer** | **Temp.** |
| --- | --- | --- | --- | --- |
| *rGapdh* | NM_017008 | tcc ctc aag att gtc agc aa | caccaccttcttgatgtcatc | 55°C |
| *rH3f3b* | NM_053985 | attcgcaagctcccctttcag | attcgcaagctcccctttcag | 51°C |
| *rRpl19* | NM_031103 | tcgccaatgccaactctcgtc | agcccgggaatggacagtcac | 54°C |
| *rSlc6a15* | NM_172321.1 | tgcatggatcaaggagaaggc | gcgacgaatgaaaacgactgg | 58.2°C |
| *mSlc6a15* | NM_175328.2 | gcatcggaagaatttctgagc | agcgacgaatgatgaacacc | 58.2°C |
| *mSlc6a17* | NM_172271.2 | ccttcatcaacttcttcacctc | cgaccacacacttctcattc | 58.2°C |
| *mMtor* | NM_020009.2 | acatttgaagaagcagag | tgatctcctccatctctt | 57.1°C |
| *mRps6* | NM_009096.3 | gagacagaagaaggaatac | tctatgccatcctacatt | 55.8°C |
| *mEif4e* | NM_007917.3 | taatcaggaggttgctaa | actggatatggttgtataga | 55.8°C |
| *mDbi* | NM_001037999.2 | caagtgggactcgtggaa | tatttcttctttagctcgtctacc | 64.5°C |
| *mβ-Tub* | NM_009451.3 | agtgctcctcttctacag | tatctccgtggtaagtgc | 55°C |
| *mRpl19* | NM_009078.2 | aatcgccaatgccaactc | ggaatggacagtcacagg | 55°C |
| *mβ-Act* | NM_007393.3 | ccttcttgggtatggaatcctgtg | cagcactgtgttggcatagagg | 55°C |
| *V73f* | - | ggcagaatacctaagagccttgt | - | 63°C |
| *J2* | - | gtccttcactgagtctggcac | - | 63°C |
| *BMP4neo1* | - | catcagagcagccgattgtc | - | 63°C |
