# Supplementary material for: B0AT2 (SLC6A15) Is Localized to Neurons and Astrocytes, and Is Involved in Mediating the Effect of Leucine in the Brain
Source: PLoS One. 2013 Mar 7;8(3):e58651. doi: 10.1371/journal.pone.0058651 (PMC3591439; doi:10.1371/journal.pone.0058651)
Supplement: Table S3 — Mouse brain expression of Slc6a15 mRNA. Scale of estimated expression; (+++) high expression, (++) medium expression, (+) low expression and (-) no apparent expression. The organization of the brain regions are described by using Franklin and Paxinos (2007) (Franklin and Paxinos, 2007). (DOCX) [file pone.0058651.s007.docx]

| **Tissue** | **Slc6a15 expression** |
| --- | --- |
| Striatum |  |
| Nucleus of the horizontal limb of the diagonal band (HDB) | +++ |
| Nucleus of the vertical limb of the diagonal band (VDB) | + |
| Accumbens nucleus, core (AcbC) | ++ |
| Caudate putamen (striatum, CPu) | +++ |
| Cerebral cortex |  |
| Layer 1 | - |
| Layer 2 | + |
| Layer 3 | ++ |
| Layer 4 | + |
| Layer 5 | +++ |
| Layer 6 | + |
| Piriform cortex (Pir) | +++ |
| Dorsal endopiriform claustrum (DEn) | ++ |
| Hippocampal formation |  |
| Granule cell layer of the dentate gyrus (GrDG) | +++ |
| Pyramidal cell layer of the hippocampus (Py) | ++ |
| Amygdala |  |
| Basolateral amygdaloid nucleus, anterior part (BLA) | +++ |
| Medial amygdaloid nucleus, posterodorsal part (MePD) | + |
| Medial amygdaloid nucleus, posteroventral part (MePV) | + |
| Lateral amygdaloid nucleus, dorsolateral part (LaDL) | ++ |
| Lateral amygdaloid nucleus, ventrolateral part (LaVL) | ++ |
| Basolateral amygdaloid nucleus, posterior part (BLP) | ++ |
| Basomedial amygdaloid nucleus, posterior part (BMP) | + |
| Posterolateral cortical amygdaloid area (PLCo) | + |
| Hypothalamus |  |
| Supraoptic nucleus (SO) | +++ |
| Paraventricular hypothalamic nucleus dorsal cap (PaDC) | ++ |
| Paraventricular hypothalamic nucleus, lateral magnocellular part (PaLM) | +++ |
| Paraventricular hypothalamic nucleus, medial magnocellular part (PaMM) | + |
| Paraventricular hypothalamic nucleus, posterior part (PaPo) | ++ |
| Anterior hypothalamic area, posterior part (AHP) | +++ |
| Anterior hypothalamic area, central part (AHC) | +++ |
| Ventromedial hypothalamic nucleus (VMH) | +++ |
| Ventromedial hypothalamic nucleus, dorsomedial part (VMHDM) | +++ |
| Ventromedial hypothalamic nucleus, central part (VMHC) | +++ |
| Ventromedial hypothalamic nucleus, ventrolateral part (VMHVL) | +++ |
| Arcuate hypothalamic nucleus (Arc) | ++ |
| Dorsomedial hypothalamic nucleus (DM) | + |
| Periventricular hypothalamic nucleus (Pe) | ++ |
| Thalamus |  |
| Zona incerta (ZI) | +++ |
| Zona incerta ventral part (ZIV) | ++ |
| Rhomboid thalamic nucleus (Rh) | ++ |
| Pons |  |
| Locus coeruleus (LC) | +++ |
| Barrington’s nucleus (Bar) | ++ |
| Cerebellum |  |
| Flocculus (FI) | + |
